# Regioisomeric Control of Chemiexcitation Efficiency in Lophine Silylperoxides through Reaction-Path Topology and Excited-State Accessibility

**DOI:** 10.1021/acsorginorgau.6c00037

**Published:** 2026-06-29

**Authors:** Diego Ulysses Melo, Andreia Boaro, Roberta Albino dos Reis, Leonardo Martins Carneiro, Paula Homem-de-Mello, Fernando Heering Bartoloni

**Affiliations:** Centro de Ciências Naturais e Humanas, 74362 Universidade Federal do ABC, Santo André, São Paulo 09210-580, Brazil

**Keywords:** chemiluminescence, lophine, peroxide, singlet quantum yield, near-degenerate states

## Abstract

This study investigates the chemiluminescence (CL) of silylperoxides derived from lophines and their associated high-energy intermediates (HEIs), specifically 1,2-dioxetanes. Three regioisomeric silylperoxides (**D1**–**3**), differing in the position of the –O^–^ group, were synthesized, and their CL and kinetic behavior under fluoride-induced decomposition were examined. Notably, the *ortho*-substituted derivative (**D1**) exhibits a 100-fold and 10-fold increase in singlet excited-state formation quantum yield compared to its *meta* (**D2**) and *para* (**D3**) counterparts, respectively. Computational analysis using density functional theory (DFT) and time-dependent DFT (TD-DFT) indicates that chemiexcitation proceeds through a single-step asynchronous-concerted mechanism with pronounced biradical character along O–O bond cleavage. The differences in singlet quantum yields are rationalized by a combined effect of the extent of the biradical/near-degenerate region and the relative accessibility of excited-state surfaces along the reaction path. In particular, the enhanced efficiency of **D1** arises from its prolonged residence in near-degenerate regions together with a more favorable energetic balance for singlet-state formation relative to triplet-state stabilization. These findings provide a mechanistic framework for understanding substituent effects in chemiexcitation and suggest that tuning reaction-path topology and excited-state energetics may guide the rational design of improved chemiluminescent systems.

## Graphical abstract

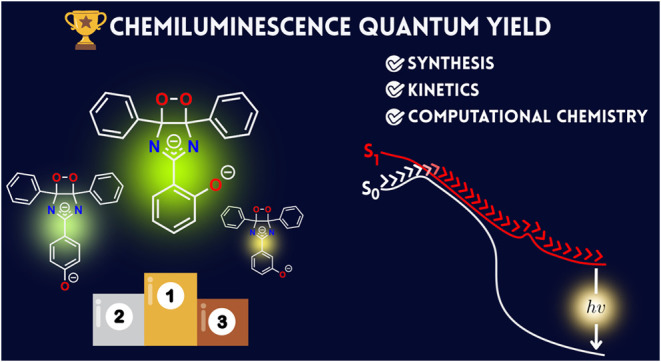

## 1 Introduction

Chemiluminescence (CL) and bioluminescence (BL) represent compelling phenomena in the field of luminescence. These processes provide unique insights into chemical reactions and biological systems capable of producing electronic excited states.^1–3^ The study of CL and BL also leads to advancements in innovative applications in areas such as analytical chemistry,^4^ environmental monitoring,^5^ imaging,^6,7^ medical diagnostics,^8,9^ and enabling technologies.^10^ Their inherent beauty and complexity make them captivating subjects for ongoing research.

Typically, every CL and BL process begins with the generation of a high-energy intermediate (HEI) from a set of reagents, in one or more steps (Figure 1A). The decomposition of the HEI produces molecules in an electronically excited state, which then deactivate through radiative decay, leading to light emission.^2,3^ Strained organic cyclic peroxides, such as 1,2-dioxetanes and 1,2-dioxetanones (Figure 1B), are known to be HEIs of many CL and BL transformations that produce light with high efficiency.^1,3,11,12^

**Figure 1:**
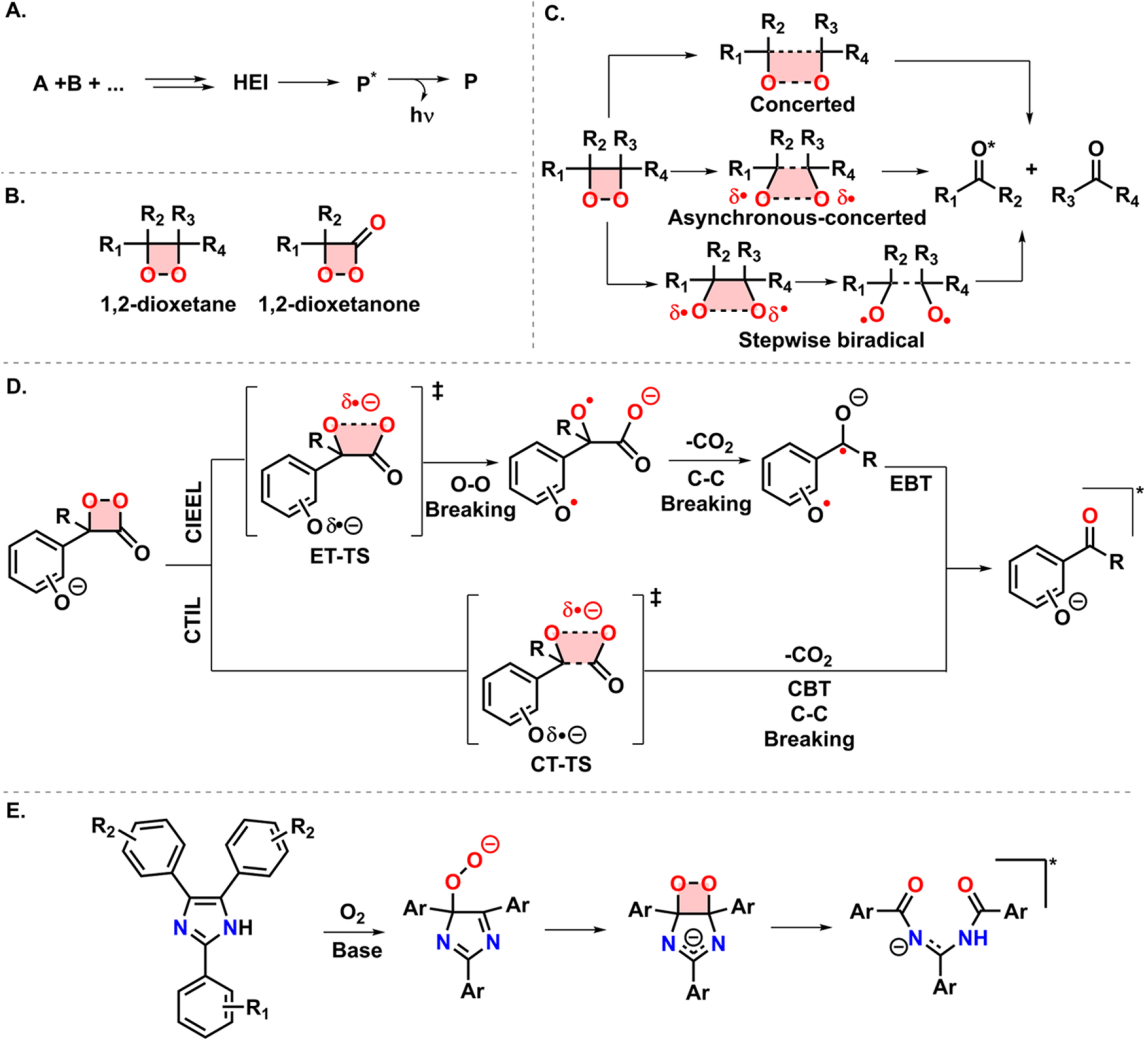
(A) General representation of the main steps involved in CL and BL processes, with generation of a high-energy intermediate (HEI) from electronic ground-state reagents (A, B, ...), further conversion of HEI to a product in the excited state (P*), and photon emission from P* generating its fundamental state (P). (B) Two classes of four-membered cyclic peroxides: 1,2-dioxetanes and 1,2-dioxetanones. (C) Decomposition of alkyl-1,2-dioxetanes (R = alkyl) through the asynchronous-concerted mechanism, proposed as a combination of two borderline pathways, the concerted (i.e., symmetry-forbidden retro-[2 + 2]-cycloaddition) and the stepwise biradical mechanisms. (D) Induced decomposition of a generic aryloxy-1,2-dioxetanone through the CIEEL and CTIL mechanisms, respectively. For a generic aryloxy-1,2-dioxetane, carbonylic compounds would be generated instead of CO_2_. (E) Oxidation of a generic lophine in basic media produces a peroxyanion that generates an anionic 1,2-dioxetane HEI *in situ*, and its decomposition leads to benzoylamidines in the excited state. Lophines are commonly prepared with substitutions at the 2-phenyl (R_1_) and 4 and 5-phenyl (R_2_) rings; peroxyanions can also be directly obtained from the deprotonation of hydroperoxides or deprotection of silylperoxides derived from lophines.

Various chemiexcitation processes exist, meaning different pathways by which a cyclic peroxide can lead to excited states. The pathway for chemiexcitation dictates the nature of the produced excited state, concerning singlet and triplet states, and also their relative quantities.

It is well-accepted that chemiexcitation with alkyl-substituted 1,2-dioxetanes occurs via an asynchronous-concerted mechanism (Figure 1C).^2,3^ This mechanism exhibits a biradical character, with the elongation of the O–O bond occurring more significantly than the breaking of the C–C bond. This model adheres to Woodward–Hoffmann rules,^13^ to the extent that it rationalizes the formation of electronically excited states, and has proven effective in explaining experimental activation parameters.^14^ Moreover, it accounts for the observation that ϕ_T_ = 0.5 E mol^–1^ and ϕ_S_ < 10^–2^ E mol^–1^ for the unimolecular thermal decomposition of 1,2-dioxetanes.^12,14–16^ A modern theoretical framework for the asynchronous-concerted mechanism posits the existence of an entropic trap during chemiexcitation.^17,18^ This entropic trap results from the redistribution of vibrational energy, facilitating the transition from the O–C–C–O dihedral angle twist—associated with O–O bond breaking—to a dissociative C–C stretch. Consequently, a greater biradical character in the decomposition of the peroxide correlates with the production of a higher yield of triplet excited states compared to singlet states, aligning with the observed experimental quantum yields.^3^

In contrast to the thermal decomposition of alkyl-1,2-dioxetanes, the CL of aryloxy-1,2-dioxetanes is associated with high ϕ_S_ values.^2,3,19^ The presence of an electron-donating substituent facilitates peroxide decomposition, and it appears that an initial intramolecular electron or charge transfer process is involved. The possible mechanistic pathways (Figure 1D) include chemically initiated electron exchange luminescence (CIEEL) and charge-transfer-induced luminescence (CTIL).

In the CIEEL pathway (Figure 1D),^20–24^ intramolecular electron transfer (ET) from the phenolate to the O–O bond generates a diradical anion intermediate. This is followed by C–C bond breaking, which yields a neutral carbonyl fragment and a formal biradical anion. Subsequently, an electron-back transfer (EBT) within the biradical anion efficiently generates singlet excited states. It is important to note that C–C bond breaking could also lead to the formation of a carbonyl radical anion and a phenoxy radical; in this scenario, the EBT would need to occur intermolecularly.^20,25,26^ However, recently more noteworthy evidence in favor of the intramolecular CIEEL pathway has been reported^2,27^

CTIL suggests that there is no need for a full-electron transfer to occur at the beginning of the sequence (Figure 1D).^28–30^ Elongation of the O–O bond leads to a charge-transfer transition state (CT-TS) and a diradical species that is immediately followed by the subsequent C–C bond breaking with simultaneous charge-back transfer (CBT). This asynchronous two-stage pathway with no discrete intermediate was proposed due to concerns about the efficiency of the EBT in CIEEL, particularly considering solvent cage effects over radical species.^29,31^ The distinction between EBT and CBT is largely formal, reflecting whether the intermediate is described as a full electron-transfer state (CIEEL) or as a charge-transfer state with incomplete charge separation (CTIL), with charge redistribution playing a central role in both mechanistic descriptions. Computational work has been done demonstrating that CTIL leads efficiently to singlet excited states with aryloxy-1,2-dioxetanones (Figure 1D), including the HEI involved in firefly BL.^28^ It is known that the position of –O^–^ in aryloxy-1,2-dioxetanes is a key determinant of chemiexcitation efficiency, ϕ_s_ is 4 orders of magnitude higher when the –O^–^ is at the *meta* position compared to *para*.^26,32^ This regioisomeric relationship of the O^–^ to the peroxidic system has also been postulated for aryloxy-1,2-dioxetanones from a CTIL perspective.^28^

The oxidation of lophine (2,4,5-triphenylimidazole) in basic media is not an efficient CL system. However, it holds historical significance as the first CL reaction ever reported.^33^ The reaction mechanism for the CL of lophine and its derivatives has been extensively studied (Figure 1E).^34–41^ Solid experimental evidence indicates that a 1,2-dioxetane is generated *in situ* as HEI from the cyclization of a peroxyanion.^36–38^ The decomposition of this HEI leads to a benzoylamidine in the excited state, with ϕ_S_ values as low as 10^–5^ E mol^–1^.^34,36–38^ Nevertheless, recent advances have demonstrated that appropriately designed lophine-based systems can achieve substantially enhanced CL efficiencies, highlighting the continuing potential of this class of compounds for practical applications.^42^ In this context, it has been demonstrated that for a –O^–^-containing derivative at the *ortho* position of the 2-phenyl ring (Figure 1E, R_1_ = −O^–^), the emission efficiency can be increased by 100-fold compared to the *meta*- and *para*–O^–^ isomers.^36^

Two noteworthy aspects of the CL of lophines are (1) its general inefficiency despite containing an electron-donating imidazolyl anion (Figure 1E) that could induce O–O bond cleavage by CIEEL or CTIL, and (2) the unusual regioisomeric effect observed for the *ortho* position. Here, we examine the CL of three regioisomeric silylperoxides **S1**-**3** derived from lophines, featuring the –O^–^ group at the *ortho*, *meta*, and *para* positions of the 2-phenyl ring. These compounds produce 1,2-dioxetanes *in situ* upon formation of a peroxyanion (Figure 1E) following fluoride-induced deprotection. In this context, we sought to evaluate whether chemiexcitation efficiency in these systems is governed by the interplay between charge availability and redistribution and the relative accessibility of the excited-state surfaces along the reaction path. We combine experimental results (kinetic and quantum yield data) and computational calculations (DFT and TD-DFT) to investigate chemiexcitation with these HEIs.

## 2 Results and Discussion

### 2.1 Synthesis and Photophysical Properties

The preparation of silylperoxides **S1**–**3** was performed using the general procedure previously reported by our group (Scheme 1).^38^ First, the lophines derivatives **L1**-**3** were prepared by condensation of their corresponding *ortho*, *meta*, and *para* hydroxyl-substituted benzaldehydes with benzil and ammonium acetate in acetic acid (Scheme 1). The hydroxyl group of **L1**-**3** was protected using *tert*-butyldimethylsilyl chloride (TBDMSCl) providing the protected derivatives **P1**–**3**, and these were then subjected to the photooxygenation reaction to obtain their respective **H1**–**3**. Finally, the peroxidic group of **H1**–**3** was also protected with TBDMS providing their corresponding silylperoxides **S1**–**3**. See the Experimental Section for more details on the preparation and characterization of these compounds.

**Scheme 1:**
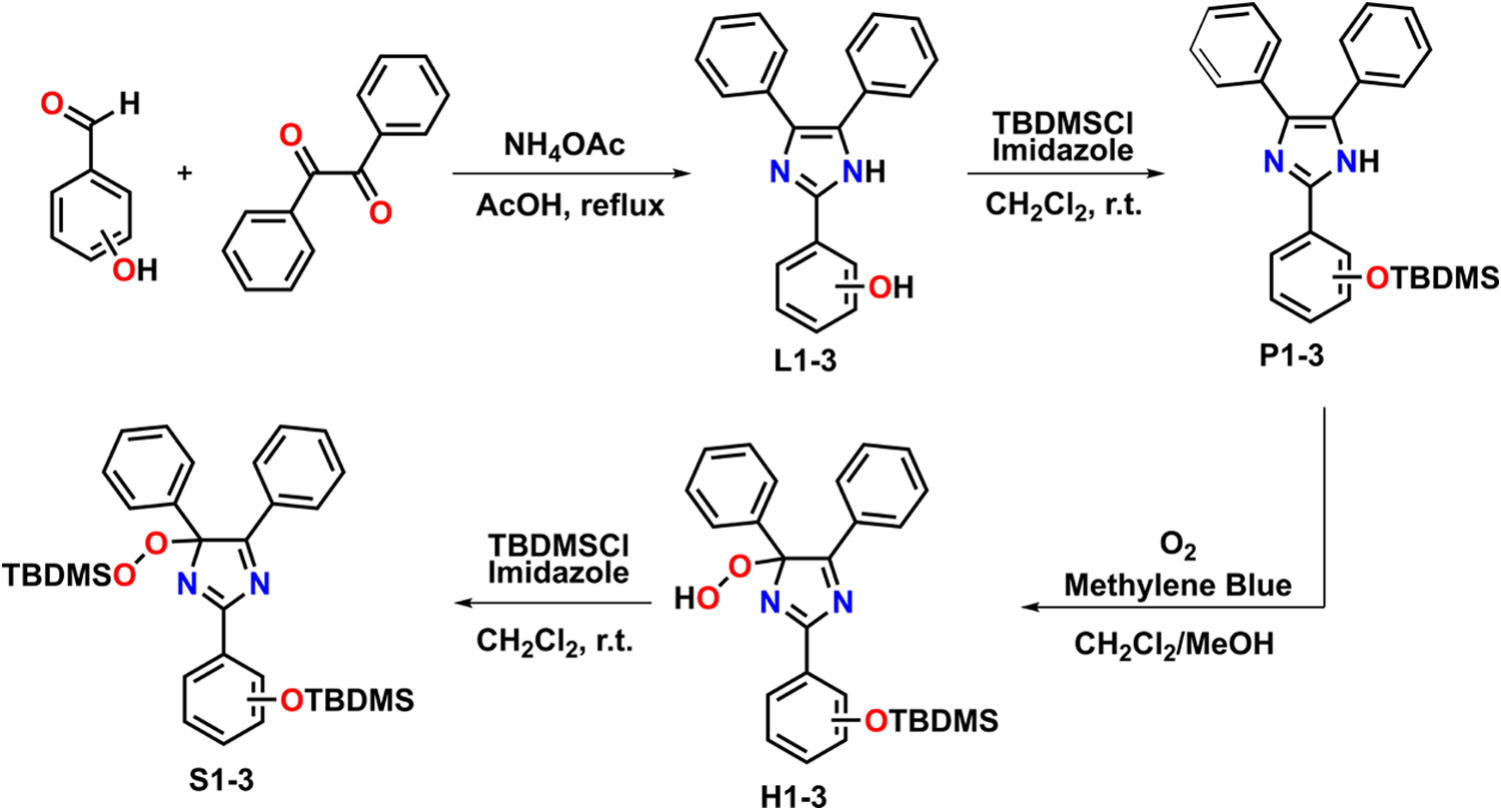
Synthetic Route for Preparing lophines **L1**-**3**, Protected Lophines **P1**-**3**, Hydroperoxides **H1**-**3**, and Silylperoxides **S1**-**3**, with the Substituent at the *ortho* (**1**), *meta* (**2**) and *para* (**3**) Positions

We compare the photophysical properties of the obtained lophines, silylperoxides, and benzoyl amidines derivatives (Table 1) with data available from the literature. As expected and reported for other lophines derivatives,^34,38,43–45^ **L1**–**3** exhibited high values of fluorescence quantum yield (ϕ_fl_), especially derivative **L2** with ϕ_fl_ above 0.4 (Table 1). The derivative containing a hydroxyl group on the *ortho* position **L1** presented a hypsochromic shift in its maximum absorption wavelength (λ_abs_) and a bathochromic shift in its maximum fluorescence wavelength (λ_fl_) compared to **L2** and **L3** (Table 1, Figure S1A,B); it also shows the highest Stokes shift value (Δ*ν*) among them: Δ*ν* = 8730, 6250, and 8020 cm^–1^, respectively, for **L1**–**3**. This occurs because the fluorescence emission of **L1** is known to be regulated by an excited-state intramolecular proton transfer (ESIPT) process involving the perturbation of keto–enol tautomerism upon photoexcitation, as reported elsewhere.^46,47^

**Table 1:** Photophysical Properties for Lophine (**L1**–**3**), Silylperoxide (**S1**–**3**), and Benzoylamidine (**B1**–**3**) Derivatives: Maximum Absorption Wavelength (λ_abs_), Maximum Fluorescence Wavelength (λ_fl_), Molar Extinction Coefficient (ϵ), Fluorescence Quantum Yield (ϕ_fl_), and Maximum Chemiluminescence Wavelength of the CL Reaction (λ_cl_)

	λ_abs_ ^a^/nm	λ_fl_ ^a^/nm	ϵ^b^/L mol^–1^ cm^–1^	ϕ_fl_ ^c^	λ_cl_ ^d^/nm
**L1**	320	444	19750 ± 30	0.34 ± 0.01	–
**L2**	309	383	28340 ± 20	0.46 ± 0.02	–
**L3**	300	395	28570 ± 80	0.35 ± 0.01	–
**S1** ^e^	279	372	16780 ± 90	(7.6 ± 0.7) × 10^–4^	550
**S2** ^e^	279	382	19120 ± 30	(4.4 ± 0.1) × 10^–3^	530
**S3** ^e^	299	391	28700 ± 70	(1.3 ± 0.1) × 10^–3^	570
**B1** ^f^	241	484	–	(7.0 ± 0.2) × 10^–2^	–
**B2** ^f^	293	382	–	0.45 ± 0.01	–
**B3** ^f^	286	394	–	(4.3 ± 0.3) × 10^–2^	–

a Obtained at 25 °C in ACN for **L1**–**3** and **S1**–**3**, and in acetonitrile:tetrahydrofuran (ACN:THF) 17:3 v/v for **B1**–**3**.

b Determined through the slope of the linear adjustment between λ_abs_ and the [**L1**-**3**] and [**S1**–**3**] with the intercept set to zero (*R* ^2^ > 0.99).

c Determined using lophine, 4-hydroperoxy-2-(4-methoxyphenyl)-4,5-diphenyl-4-imidazole, and 2,5-diphenyloxazole (ϕ_fl_ = 0.45,^48^ 0.0076,^38^ and 0.70,^49^ respectively, in EtOAc) as fluorescent standards for **L1**–**3**, **S1**–**3**, and **B1**–**3**, respectively.

d Determined for the CL reaction of [**S1**–**3**] = 0.1 mmol L^–1^ in the presence of tetrabutylammonium fluoride ([TBAF] = 1.0 mmol L^–1^) in ACN (Figure S1C).

e Absorption and fluorescence spectra are presented in Figure S1F,G.

f For **B1**–**3**, parameters λ_*abs*_ and λ_*FL*_ were determined directly from **S1**–**3** solutions after addition of TBAF (Figure S1D,E).

Compared to **L1**–**3** the silylperoxides **S1**–**3** absorb and emit light at much shorter wavelengths while being orders of magnitude less fluorescent (Table 1). This results from a heterocyclic system in **S1**–**3** that is no longer aromatic compared to the imidazolyl system in **L1**–**3**, while also not being conjugated with one of the two phenyl rings in positions 4 and 5. The λ_abs_ ca. 280–300 nm, λ_fl_ ca. 370–390 nm and ϕ_fl_ ca. 10^–4^–10^–3^ we obtained for **S1**–**3** are in agreement with values reported for other hydro- and silylperoxides derived from lophines.^38^ For the benzoylamidines **B1**–**3**, obtained upon treating **S1**–**3** with TBAF, we observed ϕ_fl_ values in agreement with former determinations (Table 1).^34,35,38^ For **B1** and **B3** the ϕ_fl_ ca. 10^–2^ is in the range observed by White and Harding for various related derivatives,^34^ for benzoylamidines substituted at the *para* position of the 2-phenyl ring,^38^ as well as derivatives substituted at the 4- and 5- phenyl rings containing a *para*-dimethylamino group at the 2-phenyl ring.^35^ For **B2** the observed ϕ_fl_ = 0.45 is comparably higher with respect to **B1** and **B3**, nonetheless, ϕ_fl_ values ca. 0.4–0.5 have been observed before for benzoylamidines containing electron-withdrawing groups at the *para* position of the 2-phenyl ring.^38^ Considering that −OH in **B2** is in the *meta* position, the electron-withdrawing character of this group by inductive effects may be taking place here to define the magnitude of its fluorescence quantum yield.

### 2.2 Chemiluminescence (CL) Reaction Overview and Kinetic Studies

The addition of fluoride (i.e., tetrabutylammonium fluoride, TBAF) to a solution containing **S1**-**3** triggers the decomposition of these peroxides, promoting the formation of excited states and observation of CL. The mechanism involved in the CL of linear peroxides (i.e., hydro- and silylperoxides) derived from lophines has been described before.^34–38^ Here we formulate it for the CL of **S1**-**3** to outline the key steps of the sequence (Scheme 2). Deprotection of the silyloxy group by F^–^ produces a peroxyanion that then cyclizes to generate the corresponding 1,2-dioxetane, **D1**-**3**. This 1,2-dioxetane is the CL process’s HEI, and electronic excited states are obtained from its decomposition (i.e., the chemiexcitation step). As with all CL and BL systems, a HEI is needed at some point to enable the generation of excited states in the chemiexcitation step.^3^ Stable alkyl- and silyloxyaryl-1,2-dioxetanes, as well as alkyl-1,2-dioxetanones,^50,51^ are considered isolable HEI that produce carbonyl fragments at the excited state.^3,12^ 1,2-Dioxetanones are involved as *in situ* HEI in the chemiexcitation step of BL systems (e.g., firefly luciferin, cipridinid, and coelenterazide, among others),^1,3^ while this class of cyclic peroxides also work as the HEI of some luciferin-analog CL reactions.^52–54^ For lophine-derived peroxides, the decomposition of the *in situ* generated 1,2-dioxetane produces benzoylamidine in the excited state, **B1**-**3** ^–*^. Finally, CL emission is observed due to **B1**-**3** ^–*^ emitting a photon and reaching the electronic ground state (S_0_), as **B1**-**3** ^–^ (Scheme 2).

**Scheme 2:**
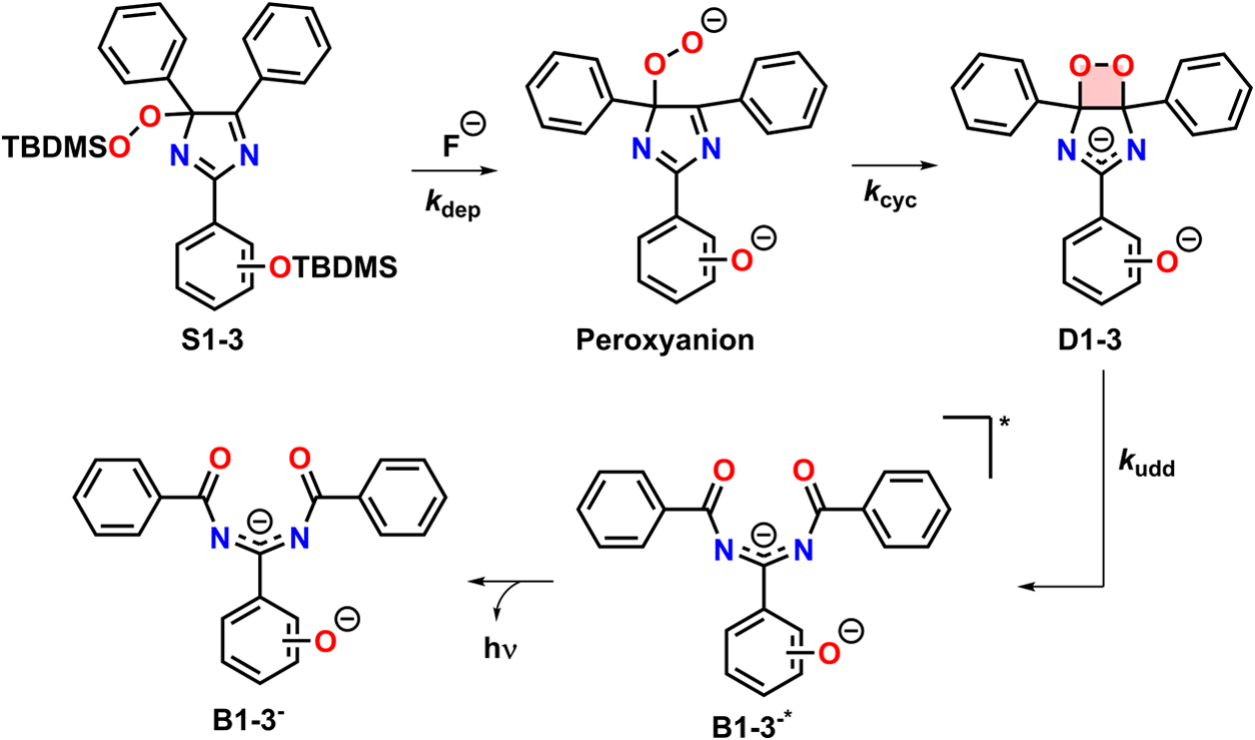
Fluoride-Triggered CL Reaction of **S1**-**3** ^a^

a The steps and their associated rate constants are discussed in the text in the context of the kinetic data.

Registering the CL spectra of **S1**–**3** after the addition of TBAF provided the maximum CL emission wavelength (λ_cl_, Figure S1C). For **S1**–**3** (0.1 mmol L^–1^) with TBAF (1.0 mmol L^–1^) in ACN/THF 17:3, we obtained λ_cl_ = 550, 530, and 570 nm, respectively. For the parent hydroperoxides **H1**-**3**, Tsunenaga, Iga and Kimura reported λ_cl_ = 564, 553, and 568 nm for their reaction with TBAF in CH_2_Cl_2_/THF 5:1.^36^ Lu and collaborators studied two isomeric silylperoxides closely related to **S1**-**3** and observed the CL emission ca. 560 nm in CHCl_3_ or acetone, also with TBAF.^37^ For 11 different hydro- and silylperoxide derivatives derived from lophines, it was reported that λ_cl_ = 500–530 nm.^38^ Lastly, White and Harding observed that CL occurs ca. 500–530 nm for the direct oxidation of various lophines in strongly basic media; likewise, they observed similar emission wavelengths for some hydroperoxides.^34^ It is clear from all these studies that the emitting species of the ongoing CL reaction is the benzoylamidine anion. Our data are consistent with that emission arises from **B1**-**3** ^–*^ generated after deprotecting **S1**-**3** with F^–^ (Scheme 2). It should be highlighted that the observed λ_fl_ for **B1**-**3** (Table 1) is not equivalent to the λ_cl_ of the reaction we studied (see also Figure S1 for the respective spectra). Although, in general, it is expected that the λ_fl_ of a proposed emitter matches exactly the λ_cl_ of the reaction, such disparity in wavelengths has been consistently observed for the CL of lophines^34^ and their linear peroxides.^36,38^ One proposed hypothesis^34,36^ is that emission originates from a conformer of the benzoylamidine anion in the excited state which is not the one related to S_1_/S_0_ deactivation through typical fluorescence; this is a relevant aspect for the discussion we will present later in this work.

To study the kinetics of the reaction, we observed the CL intensity-time profiles obtained from the TBAF-induced decomposition of **S1**–**3** using a commercial test tube luminometer in ACN:THF 17:3 v/v, under pseudo-first order conditions for peroxides. The obtained CL intensity-time profiles are very reproducible and consist of a rapid CL increase after the addition of TBAF to a **S1**–**3** solution followed by a slower fall (Figure S2). Our research group, while studying the CL of hydro- and silylperoxides derived from lophines,^38^ proposed a mechanistic interpretation of the CL intensity-time profiles, ultimately concluding that a 1,2-dioxetane is involved as the HEI of the system. We apply the same interpretation for the kinetic data obtained with the CL of **S1**-**3** to determine the rate constants for *k*_dep_ and *k*_cyc_, for the first and second steps of the reaction (Scheme 2).

The rate constant for the deprotection of **S1**–**3** (*k*_dep_, Scheme 2) is obtained from the linear dependence of the observed rate constant for the fall in CL intensity (*k*_obs_^1^, Tables S1–S3) with fluoride concentration (Figure S2). We obtained *k*_dep_ = (1.54 ± 0.03) × 10^3^, (1.95 ± 0.02) × 10^3^, and (2.70 ± 0.03) × 10^3^ L mol^–1^ s^–1^, respectively, for **S1**-**3**. These are in agreement with values previously reported for silylperoxides *para*-methoxy and *para*-methyl-substituted in the 2-phenyl ring, with *k*_dep_ = 1.30 × 10^3^ and 2.13 × 10^3^ L mol^–1^ s^–1^, respectively.^38^ We assume that the silyloxy group has already been converted to phenolate when the silylperoxide group is deprotected, given that peroxyanions are less stable as conjugate bases than phenolates.^55^ The deprotection reaction rate appears to be related to the distance between the peroxide and phenolate anions, as greater distances result in higher values of *k*_dep_, although the observed difference is modest.

From the linear dependence between the observed rate constant for the rise in CL intensity (*k*_obs_^2^, Tables S1–S3) and the lowest TBAF concentrations, the *k*_cyc_ values for the HEI formation reaction were determined for **S1**–**3** (Scheme 2, Figure S4). Similar rates were obtained for **S1** and **S2**, *k*_cyc_ = (4.8 ± 0.1) × 10^4^ and (4.5 ± 0.1) × 10^4^ L mol^–1^ s^–1^, respectively, being about six-times higher than the value previously reported by our research group for the silylperoxide derivative substituted with *para*-methoxy (*k*_cyc_ = 0.76 × 10^4^ L mol^–1^ s^–1^).^38^ For the *para*-substituted derivative **S3**, the obtained *k*_cyc_ = (12.8 ± 0.6) × 10^4^ L mol^–1^ s^–1^ cyclization rate constant is slightly higher compared to **S1** and **S2**. Although the formation of the 1,2-dioxetane HEI from the cyclization of the peroxyanion is unimolecular, *k*_cyc_ has bimolecular units. This is because the generation of HEI depends on the concentration of the peroxyanion, which is defined by the concentrations of silylperoxide and fluoride. This dependency reflects an intrinsic aspect of the kinetics of complex reactions,^56^ as previously discussed for other CL systems involving an anion generated by the action of a Lewis base.^38,57^ Our experimental kinetic data clearly show that **D1**-**3** is generated as an intermediate in this CL reaction, following the same mechanism previously discussed by our group.^38^

### 2.3 Chemiluminescence and Singlet Excited States Formation Yields

Intensity-time profiles obtained from the decomposition of **S1**–**3** induced by different TBAF concentrations (Figure S2) were integrated and the CL quantum yield (ϕ_cl_) was determined for each TBAF concentration; see Experimental Section for details. ϕ_cl_ is independent of the fluoride concentration and mean values were obtained for each silylperoxide (Table 2). Constant values of ϕ_cl_ indicate that, under the studied experimental conditions, the formation yield of the 1,2-dioxetane HEI is constant. Moreover, fluoride does not act as a quencher of the emitting excited electronic states. The ϕ_cl_ values in the order of 10^–6^ E mol^–1^ obtained for **S2** and **S3** are consistent with the ones previously reported for analogous silylperoxides^37,38^ and hydroperoxides^34,35^ derived from lophines. This CL reaction presents low light emission efficiency compared to other CL reactions, such as the peroxyoxalate reaction^45,49^ and the induced decomposition of aryloxy-1,2-dioxetanes,^32^ which can reach ϕ_cl_ = 0.1 up to 0.6 E mol^–1^. Nevertheless, the *ortho*-substituted derivative **S1** exhibited ϕ_cl_ = 1.6 × 10^–4^ E mol^–1^, 2 orders of magnitude higher compared to **S2** and **S3** (Table 2).

**Table 2:** Chemiluminescence Quantum Yield (ϕ_cl_) and Singlet Excited State Formation Quantum Yield (*ϕ*_s_) for the CL Decomposition Reaction of **S1**-**3** Induced by Fluoride^a^

Compound	ϕ_cl_/10^–6^ E mol^–1^	ϕ_s_/10^–5^ E mol^–1^
**S1**	160 ± 20	220 ± 30
**S2**	6.9 ± 0.5	1.5 ± 0.1
**S3**	7.4 ± 2.6	17 ± 6

a The [**S1**] = 0.127 μmol L^–1^ and [**S2**-**3**] = 1.27 μmol L^–1^, the [TBAF] was varied from 0.037 to 0.210 mmol L^–1^ (see Tables S1–S3 for details).

The singlet excited states formation quantum yield (ϕ_s_) was determined for **S1**–**3** (Table 2) by correcting ϕ_cl_ by the fluorescence quantum yield (ϕ_fl_) of the emitting species **B1**-**3** ^–^ (Table 1); see Experimental Section for details. Each silylperoxide has a different magnitude of ϕ_s_, with the *ortho*-substituted derivative **S1** exhibiting the highest ϕ_s_ value, i.e., 2.2 × 10^–3^ E mol^–1^, followed by the *para* and *meta*-substituted derivatives (Table 2). Tsunenaga, Iga and Kimura reported that the relative emission intensity of the fluoride-induced CL of hydroperoxide **H1** is at least 2 orders of magnitude higher than the relative intensity observed for **H2** and **H3**.^36^ These authors reported relative CL intensities for the isomeric hydroperoxides **H1**-**3** to discuss the difference in their chemiexcitation efficiency, while we report absolute ϕ_s_ values for **S1**-**3**; nevertheless, our results are in agreement with their observation. In the following section, we present the calculated data we obtained for the decomposition pathway of **D1**-**3**, to then use it to rationalize the observed differences in the experimental ϕ_s_ values for the CL of **S1**-**3**.

### 2.4 DFT and TD-DFT Computational Study

To better understand the chemiexcitation mechanism involved in the decomposition of high-energy intermediates, specifically, 1,2-dioxetanes generated *in situ* by the fluoride-triggered reaction of **S1**-**3** (Scheme 3) we performed density functional theory (DFT) and time-dependent density functional theory (TD-DFT) calculations. We acknowledge that multiconfigurational and multireference quantum-chemical methods (e.g., MS-CASPT2) are highly beneficial for describing bond-breaking processes that possess biradical character and involve strong electronic-state mixing, as demonstrated in previous studies with homolytic O–O bond cleavage in peroxide-like systems.^58,59^ However, the lophine derivatives investigated in the present work are relatively large molecular systems, highly conjugated, and containing up to 40 atoms. This makes conventional wave function-based multiconfigurational methods computationally prohibitive, particularly when the exploration of potential energy surfaces (PES) and the comparative analysis of several derivatives are required. Consequently, we adopted unrestricted DFT and TD-DFT combined with the broken-symmetry formalism and an implicit solvent model as practical alternatives. These methods provide a reasonable balance between computational cost and accuracy for investigating energetic trends and have been successfully employed in previous studies to describe chemiexcitation processes.^60–62^

**Scheme 3:**
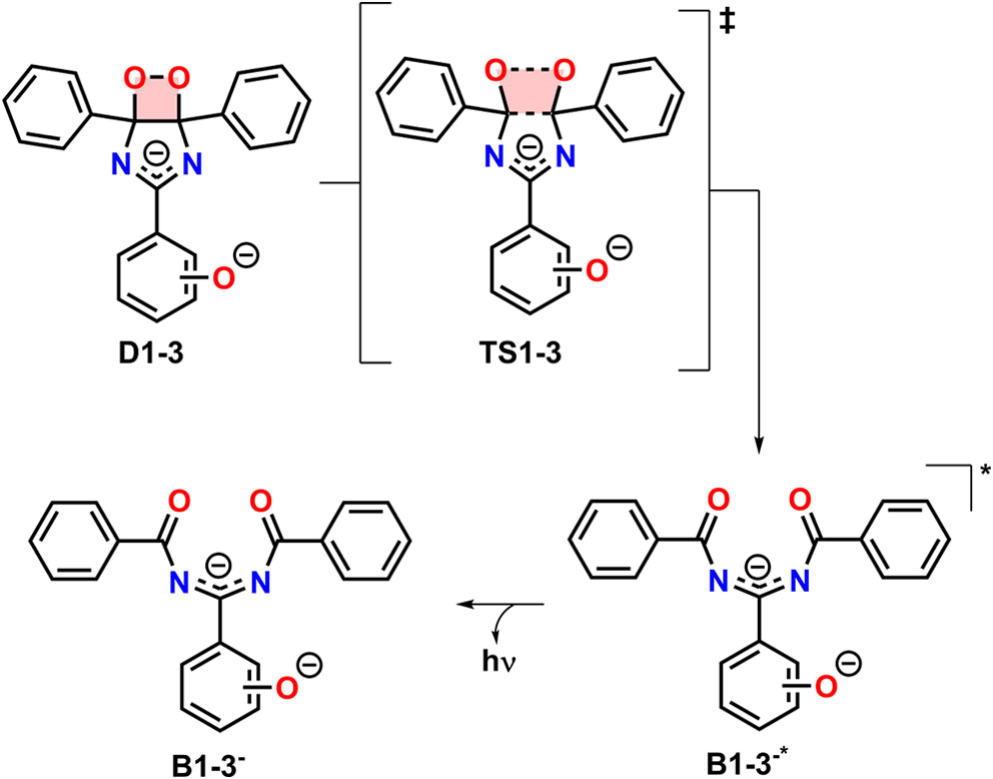
Chemiexcitation Pathway for Decomposition of **D1**-**3** to **B1**-**3** ^–*^, Ultimately Leading to Light Emission and Ground-State Products^a^

a The scheme includes a representation of the inspected transition state (**TS1**-**3**).

In this work, we obtained the optimized geometry of high-energy intermediate 1,2-dioxetanes (**D1**-**3**, Scheme 3), transition states (**TS1**-**3**, Scheme 3) at ground state (S_0_), as well the most probable reaction product and emitting species, benzoyl amidine at S_0_ (**B1**-**3** ^–^, Scheme 3). Additionally, we performed an intrinsic reaction coordinate (IRC) analysis at S_0_, and TD-DFT for the S_1_ and first triplet excited state (T_1_) to confirm the validity of the TS structures and access geometric and electronic information along the potential energy curve (PEC) of the decomposition reaction of **D1**-**3**.

For both the **D1**-**3** and **B1**-**3** ^–^ derivatives, we applied closed-shell RKS CAM-B3LYP/cc-pVTZ//CPCM­(acetonitrile) level of theory for geometry optimization. At the same time, considering that a closed-shell does not accurately reproduce the decomposition mechanism of cyclic peroxides,^63^ we used open-shell UKS at the same level of theory combined with the broken symmetry (BS) technique for TS and IRC calculations. We performed vibrational analysis for all optimized geometries, i.e., stationary points and TSs, to confirm the absence and presence of a single imaginary frequency, respectively. Long-range corrected density functionals,^64,65^ especially the CAM-B3LYP, were successfully used to investigate the decomposition of other cyclic peroxides in chemiluminescent and bioluminescent systems.^61,62,66–68^

In the PEC from IRC data, we can track the breaking of the O–O and C–C bonds, as well as the changes in the O–C–C–O dihedral angle along the reaction pathway (Figure 2); the relevant geometric information about the **D1**-**3**, **TS1**-**3** and **B1**-**3** ^–^ is present in Table S4. For **D1**-**3**, the O–O and C–C bond lengths obtained here are consistent with those reported for AMPPD 1,2-dioxetane and without apparent influence from the substituent position in O–O and C–C bond lengths, and O–C–C–O dihedral angle.^66^ We observed a single TS for the decomposition for all derivatives **D1**-**3**. These decomposition of **D1**-**3** begins with the elongation of the O–O bond, an increase in the dihedral angle, and a slight variation in the C–C bond length until the TS is reached (Figure 2); specifically at TS, the expected value of the spin operator (⟨S^2^⟩) shows a biradical characteristic, i.e., ⟨S^2^⟩ equal to 0.32, 1.03, and 0.43 for **TS1**-**3**, respectively. After the TS, the O–O bond continues to elongate, then we observe an initial elongation of the C–C bond when the O–O bond reaches 2.264, 2.128, and 2.240 Å for **TS1**-**3**, respectively (Figure 2); thus indicating that the O–O and C–C bond breaks occur asynchronously. Additionally, the TS is characterized by a large imaginary frequency (979.1*i*, 1004.8*i* and 978.1*i* cm^–1^ for **TS1**-**3**, respectively), which mainly corresponds to the stretching of peroxide O–O bond, following the result that shows O–O bond elongation from **D1**-**3** conducts the process to the TS. The trend in the geometrical parameters variation we observe here, specifically the asynchronous process and absence of a transition state relative to C–C bond break, is consistent with the reported decomposition of anionic cyclic peroxides involved in the efficient bioluminescence mechanism.^67^

**Figure 2:**
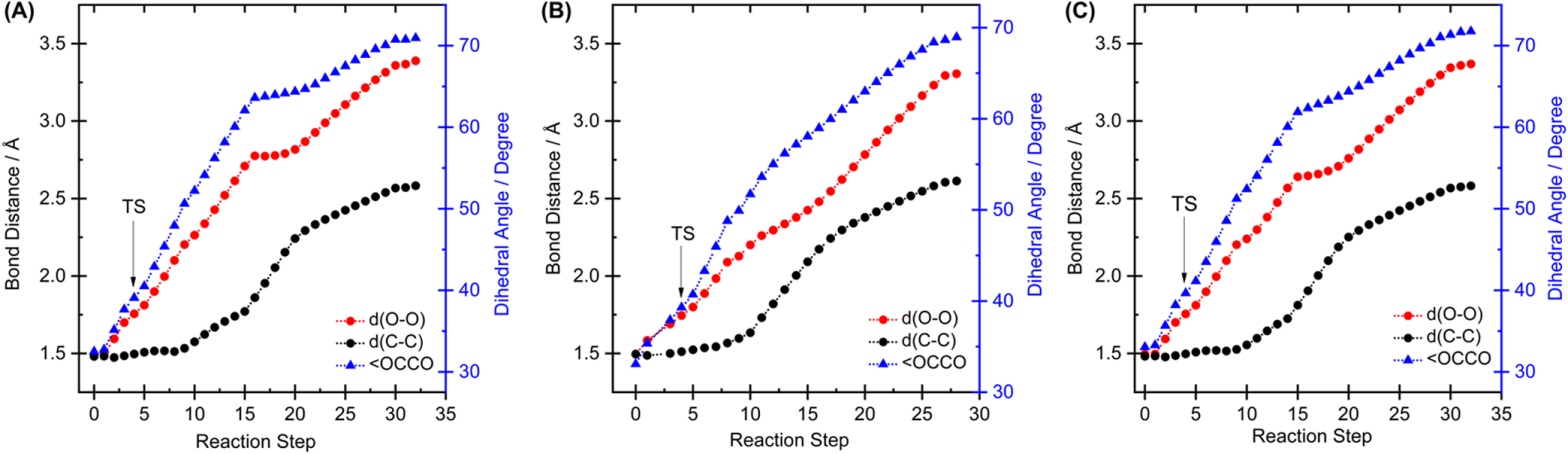
Variation of O–O and C–C bond lengths and O–C–C–O dihedral angles along reaction path of (A) **D1**, (B) **D2**, and (C) **D3** decomposition calculated by (BS)­UCAM-B3LYP//cc-pVTZ//CPCM­(acetonitrile) level of theory.

From these same optimized geometries and thermochemistry derived from vibrational frequency calculation, we can also extract related thermodynamic information, such as the Gibbs free energy (Δ*G*_rx_) and the enthalpy (Δ*H*_rx_) of the reaction, as well as the Gibbs free energy (Δ*G* ^‡^) and enthalpy (Δ*H* ^‡^) of the activation for the decomposition of **D1**-**3** (Table 3). The values Δ*G*_rx_ and Δ*H*_rx_, both lower than −160 kcal mol^–1^ show a spontaneous highly exergonic and exothermic reactions. The values reported here for Δ*G* ^‡^ and Δ*H* ^‡^ are both within the range of 9–10 kcal mol^–1^, and specifically the Δ*G* ^‡^ follows the reported computational values from the decomposition of anionic 1,2-dioxetanones (ca. 10 kcal mol^–1^),^60^ and are reasonable within experimental values from the catalyzed decomposition of 1,2-dioxetanes (ca. 20 kcal mol^–1^).^24,32^

**Table 3:** Gibbs Free Energy (Δ*G*_rx_) and the Enthalpy (Δ*H*_rx_) of the Reaction, Gibbs Free Energy (Δ*G* ^‡^) and Enthalpy (Δ*H* ^‡^) of the Activation for the Decomposition of **D1**-**3^a^**

Compound	Δ*G*_rx_	Δ*H*_rx_	Δ*G* ^‡^	Δ*H* ^‡^
**D1**	–167.5	–166.0	9.8	9.0
**D2**	–166.9	–164.5	10.5	9.8
**D3**	–170.8	–168.4	8.9	8.8

a Values in kcal mol^–1^.

Given the ongoing debate about the biradical nature and the mechanism involved in 1,2-dioxetane decomposition, we further analyzed ⟨S^2^⟩ along the PEC. Figure 3A–C shows the reaction change from the closed-shell state (⟨S^2^⟩ = 0) to the open-shell biradical singlet state (⟨S^2^⟩ ca. 1) as it approaches the TS. This biradical characteristic remains after TS for all derivatives **D1**-**3** until reaching 76%, 52%, and 70% relative reaction progress for **D1**-**3** (gray areas in the Figure 3A–C). At the TS, the spin density at the two oxygen atoms from the peroxide (inset Figure 3A–C) allows us to assign that biradical structure arises from the homolytic O–O bond break without an apparent electron transfer, as expected from the decomposition of cyclic peroxides by the intramolecular CIEEL mechanism.

**Figure 3:**
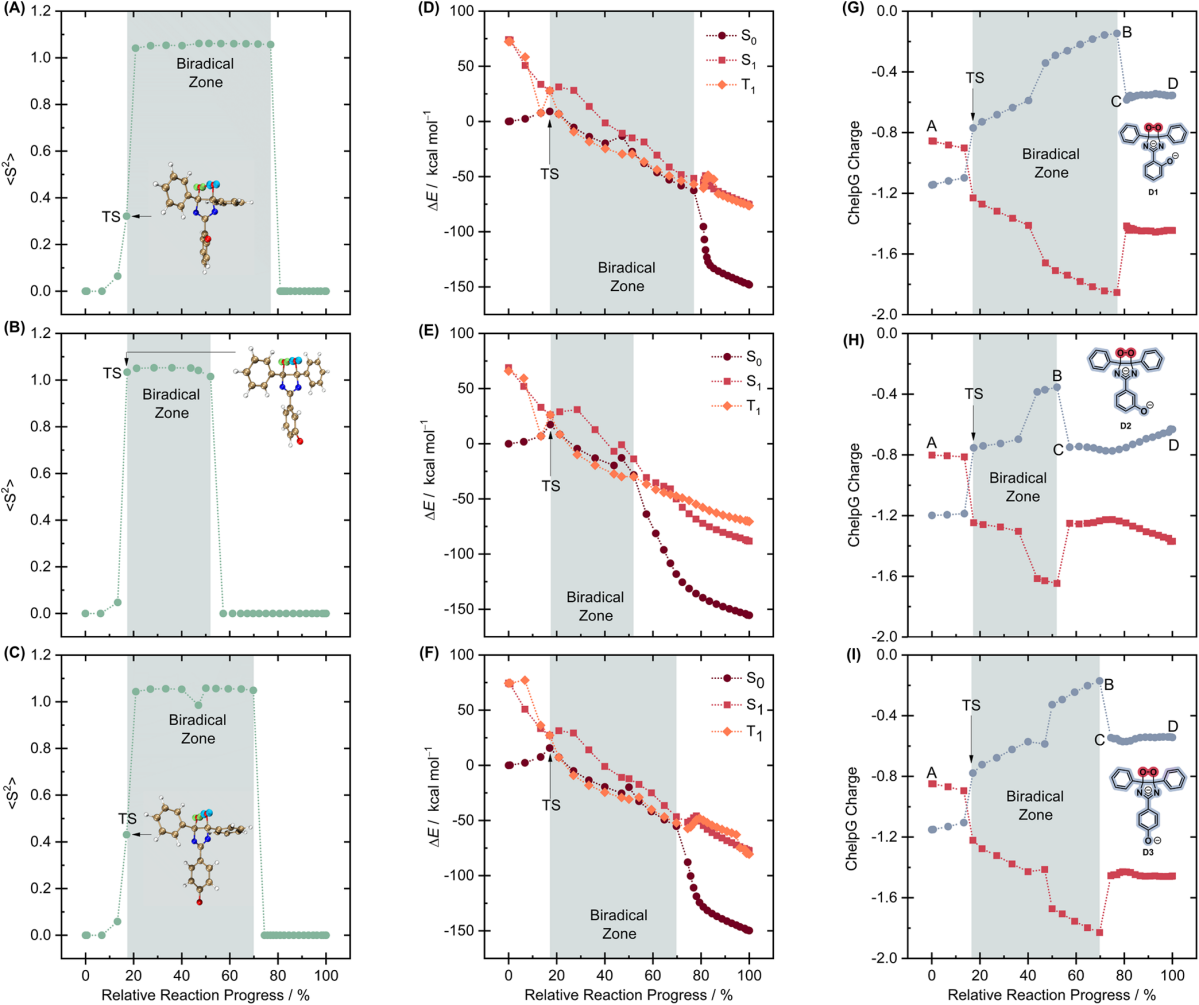
Expected of the spin operator (⟨S^2^⟩) value along the relative reaction progress of (A) **D1**, (B) **D2**, and (C) **D3** (the inset molecules are the spin density plot at the transition state). The potential energy surface (PEC) of S_0_, S_1_ and T_1_ states along the relative reaction progress of (D) **D1**, (E) **D2**, and (F) **D3**. The population of ChelpG charges on oxygens peroxide atoms and the rest of the molecule at S_0_. All calculated by (BS)­UCAM-B3LYP//cc-pVTZ//CPCM­(acetonitrile) level of theory. The gray area represents the biradical zone, and the relative reaction progress scale was built considering the variation of the dihedral angle across the obtained IRC curve.

Moving to analyze the energy as a function of relative reaction progress (Figure 3D–F), the reaction initiates with the increase of Δ*E* for **D1**-**3** until the TS was located at approximately 17% relative reaction progress. After this point, the Δ*E* decreased smoothly (by around −50 kcal mol^–1^ for **D1** and **D3**, and −25 kcal mol^–1^ for **D2**) until reaching 76% for **D1**, 52% for **D2**, and 70% for **D3**, then Δ*E* abruptly decreased from these points until it reached the reaction product. As illustrated in the gray area in the Figure 3D,F, from the TS for all derivatives, the PECs for S_0_, S_1_ and T_1_ states approach each other along the relative reaction coordinate, an entropic trap region,^18^ with an average S_1_–S_0_ energy gap of 17.1, 18.5, and 17.4 kcal mol^–1^ for **D1**-**3**, and average T_1_-S_0_ energy gap of 0.4, −4.2, and −0.6 kcal mol^–1^ for **D1**-**3**. This region allows the intersystem crossing (ISC) from singlet and triplet states; however, considering the gaps S_1_–S_0_ and T_1_–S_0_, if the system reaches excited states, these results indicate that the formation of product in the nonemissive T_1_ state could be dominant. Interestingly, the biradical zone coincides with degenerated or near degenerated states, and after escape from this zone, the degenerescence of ground states with the excited states vanishes. These PECs also show that the different substituents remain in this biradical zone and degenerate or near degenerate states to different extents for the relative reaction progress, 60%, 35% and 53% for **D1**-**3**, respectively; this behavior can be used to rationalize the experimental efficiency in the singlet excited states generation in the decomposition of **D1**-**3** (Table 2), i.e., ϕ_s_(**D1**) > ϕ_s_(**D3**) > ϕ_s_(**D2**).

The order of ϕ_s_ for **D1**-**3** can be understood as the result of a combined effect of the extent of the biradical/near-degenerate region and the relative accessibility of the excited-state surfaces along the reaction path.^17,18^ **D1** remains in this region for the largest fraction of the reaction coordinate, whereas **D2** and **D3** do so for progressively shorter extents. In addition, **D1** exhibits the smallest average S_1_–S_0_ gap among the three derivatives, while its average T_1_-S_0_ gap is less favorable for triplet stabilization than those of **D2** and **D3**.^3,61,62,67,68^ Taken together, these features make singlet-state formation comparatively more favorable for **D1**, intermediate for **D3**, and least favorable for **D2**, in agreement with the experimental order ϕ_s_(**D1**) > ϕ_s_(**D3**) > ϕ_s_(**D2**). Although the relative energetics suggest that triplet-state formation may compete with singlet-state formation, quantitative assessment of the associated intersystem crossing kinetics would require explicit evaluation of spin–orbit and nonadiabatic couplings, which were not considered in the present study. Collectively, these observations indicate that tuning the extent of near-degenerate regions and the accessibility of excited-state surfaces may offer a useful framework for the rational design of new chemiluminescent systems. A conceptual summary of the factors governing the observed regioisomeric dependence of chemiexcitation efficiency is presented in Figure 4.

**Figure 4:**
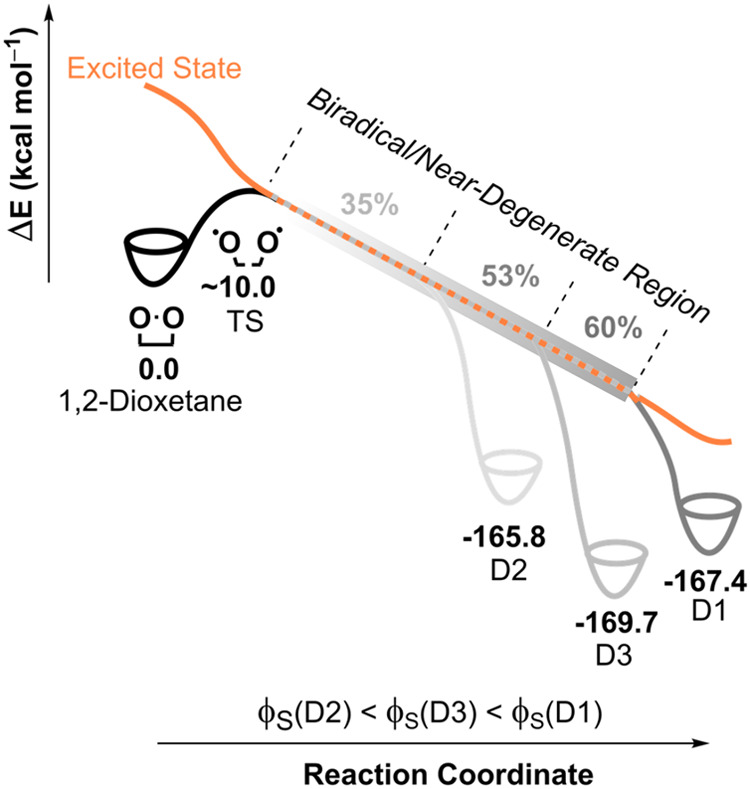
Conceptual summary of the regioisomeric control of chemiexcitation efficiency in **D1**–**3**. The experimentally observed order for singlet excited states generation yield is rationalized by the combined effect of the extent of the biradical/near-degenerate region along the reaction coordinate and the relative accessibility of excited-state surfaces. The percentages indicate the fraction of the reaction coordinate associated with the near-degenerate region.

Considering the importance of charge and electron transfer, as well as the BCT and BET, in the chemiexcitation process, we investigate the electron population along the PEC. Considering the smaller dependence of the electrostatic potential on a grid (ChelpG) with the level of theory compared with other charge schemes,^69^ we investigate the decomposition of **D1**–**3** applying this scheme. Figure 3G–I illustrates the population analysis by ChelpG along the PEC at S_0_, and Table S5 accounts for values in highlighted points. To enhance clarity in visualizing the electronic processes during the reaction, the molecules **D1**–**3** were divided into two distinct regions: one comprising the oxygen atoms of the peroxide group and the other representing the rest of the molecule, as shown in the insets of Figure 3G–I. From point A, the reaction begins with a charge transfer (CT) directed toward the oxygen atoms in the peroxide group, and this CT becomes more significant after the TS and in the biradical zone. This results in a full electron transfer at point B on the PEC for derivatives **D1** and **D3** (Figure 3G and I), i.e., Δ*q ca.* 1*e*. In contrast, compound **D2** (Figure 3H) only undergoes partial CT, Δ*q* equal to 0.845*e*, never achieving a full electron transfer. As the reaction progresses past the biradical zone toward point C, a reverse CT is observed for all compounds, Δ*q* equal −0.439, −0.395 and −0.374*e* for **D1**-**3**, respectively, where the charge is transferred from the oxygen atoms of the peroxide group back to the rest of the molecule.

On the other hand, when we investigate the charges along the PEC at S_0_ using the Mulliken scheme (Figure S5), for all **D1**–**3**, we observe only a CT (0.551–0.617*e*) and a BCT (0.257–0.286*e*) in the same points at the relative reaction progress of the population of ChelpG charges. These values are in line with the thermolysis of 1,2-dioxetane and 1,2-dioxetanone described with CTIL,^28^ and a slightly modified CTIL mechanism, the gradually reversible CT initiated luminescence (GRCTIL) mechanism.^67^ Although charge redistribution is clearly present along the reaction coordinate, these results suggest that it does not, by itself, account for the observed differences in chemiexcitation efficiency. Atomic charge analyses remain a common tool for discussing charge involvement in CL and BL mechanisms,^2,3^ and the development and application of complementary charge-partitioning and electronic-structure descriptors may further refine mechanistic interpretations in systems where charge redistribution, biradical character, and state crossings occur simultaneously.^69^

## 3 Conclusion

This study presents a combined experimental and computational investigation of the chemiluminescence of silylperoxides **S1**–**3** derived from lophines and their associated high-energy intermediates, the 1,2-dioxetanes **D1**–**3**. Kinetic analysis of the fluoride-induced decomposition of **S1**–**3** provided rate constants relevant to HEI formation and decay, while experimental quantum yields (ϕ_cl_ and ϕ_s_) revealed that the *ortho*-O^–^ derivative is 100-fold and 10-fold more efficient in producing excited states than its *meta* and *para* regioisomers, respectively. Computational calculations (DFT and TD-DFT) indicate that chemiexcitation proceeds through a single-step asynchronous-concerted pathway with pronounced biradical character along O–O bond cleavage. The differences in singlet quantum yields are rationalized by a combined effect of the extent of the biradical/near-degenerate region and the relative accessibility of excited-state surfaces along the reaction path. In this context, **D1** benefits from both a longer residence in near-degenerate regions and a more favorable energetic balance for singlet-state formation relative to triplet-state stabilization, whereas **D2** and **D3** exhibit progressively less favorable combinations of these factors. These results support the view that chemiexcitation efficiency is governed by the interplay between reaction-path topology and excited-state energetics, rather than by a single mechanistic factor, with charge availability and redistribution playing a secondary role. In this sense, controlling the extent of near-degenerate regions and the accessibility of excited-state surfaces may provide a useful framework for the rational design of new chemiluminescent systems. Furthermore, the integration of computationally guided structural modifications with experimental validation may offer a promising strategy for the development of more efficient emitters in chemiluminescent applications. While the present analysis provides a consistent mechanistic interpretation, it is based on a limited set of regioisomeric derivatives and computational approaches that do not explicitly account for all nonadiabatic effects. Further studies combining a broader range of systems with more advanced electronic-structure methods may help to assess the generality of these findings and refine the mechanistic framework proposed here.

## 4 Material and Methods

### 4.1 Experimental Section

#### 4.1.1 General Procedures and Equipment

All chemical reagents and solvents with analytical grade used in this work were purchased from commercial sources and used without further purification. All reported CL parameters were obtained as mean value ± standard deviation for at least three replicas at the same experimental condition. The purity of the silylperoxides used in kinetic measurements (over 97% in mass) was determined by iodometry using the method of spectrophotometric quantification of I_3_^–^.^50,70–72^

Nuclear magnetic resonance (NMR, ^1^H and ^13^C) spectra were recorded at 25 °C using a 500 MHz Varian spectrometer belonging to the Multiuser Central Facilities at UFABC (CEM-UFABC). Elemental analyses were carried out on a Thermo Scientific Flash EA 1112 analyzer belonging to CEM-UFABC. Organic solvents were removed by low-pressure distillation using a rotary evaporator Büchi R-215 coupled to the V-855 vacuum controller, the V-700 membrane pump, the F-108 recirculating refrigerator, and the B-491 heating bath. For UV–vis absorption and fluorescence emission assays, the Varian Cary 60 and Varian Cary Eclipse spectrophotometers were used, respectively, using quartz cuvettes for absorption and fluorescence with a maximum volume of 3.0 mL and an optical path of 10.0 mm. Both equipment cell holders were maintained at 25 °C using a Varian Cary PCB 1500 system. Chemiluminescence assays were performed in a Berthold Sirius single-tube luminometer with a set of automatic injectors. Deionized water was obtained through a Milli-Q Millipore purifying system (conductivity 18.2 MΩ cm^–1^).

#### 4.1.2 Lophines L1–3 Preparation

##### 4.1.2.1 Lophines L1–3 Preparation

The preparation of lophines **L1**–**3** was carried out based on the method previously used by our group,^38,45,73^ inspired by Benisvy et al.^74^ A 100 mL round-bottom flask, equipped with a reflux condenser and magnetic stirring, was charged with 12 mmol (1 equiv) of benzil, 96 mmol (8 equiv) of ammonium acetate, 50 mL (0.24 mol L^–1^) of glacial acetic acid, and 13.2 mmol (1.1 equiv) of the respective substituted benzaldehydes, i.e., of salicylaldehyde, 3-hydroxybenzaldehyde, and 4-hydroxybenzaldehyde. The resulting mixture was kept under reflux and magnetic stirring for 4 h at 170 °C, using an oil bath. After that, 100 mL of cold deionized water was added to induce the product precipitation. The crude product was filtered, washed with cold water, and dried under vacuum (10^–3^ mbar, 30 min). Such solid was solubilized in 300 mL of ethyl acetate (EtOAc) and magnesium sulfate was added to retain residual water. Then, the solution was filtered and the solvent was removed by rotary evaporation and under vacuum for 30 min. Purification was performed by recrystallization with EtOAc, and the pure cotton wool-like crystals were filtered under vacuum, washed with cold pentane or hexane (Hex), and dried under vacuum for 3 h. **L1–3** were characterized by NMR (^1^H and ^13^C, Figures S5–S7) and EA.

##### 4.1.2.2 2-(4,5-Diphenylimidazol-2-yl)­phenol (L1)

2.72 g (67%), colorless amorphous solid, *R*_f_ = 0.59 (SiO_2_, Hex/EtOAc 4:1). Anal. Calcd for C_21_H_16_N_2_O: C, 80.7; H, 5.2; N, 9.0; O, 5.1. Found: C, 80.4; H, 5.2; N, 8.8. ^1^H NMR (Acetone-d_6_, 500 MHz): δ 12.88 (s, 1H), 7.97 (dd, 1H, *J* = 7.8, 2.8 Hz), 7.63–7.57 (m, 4H), 7.48–7.35 (m, 3H), 7.31–7.28 (m, 2H), 7.02–6.92 (m, 2H) ppm. ^13^C­{^1^H} NMR (Acetone-d_6_, 125 MHz): δ 171.0, 164.1, 158.7, 147.3, 136.0, 135.1, 131.7, 131.1, 129.7, 129.6, 129.3, 129.2, 128.2, 128.0, 125.4, 119.7, 118.1, 114.0 ppm.

##### 4.1.2.3 3-(4,5-Diphenylimidazol-2-yl)­phenol (L2)

3.92 g (97%), colorless amorphous solid, *R*_f_ = 0.66 (SiO_2_, Hex/EtOAc 1:1). Anal. Calcd for C_21_H_16_N_2_O: C, 80.7; H, 5.2; N, 9.0; O, 5.1. Found: C, 80.6; H, 5.2; N, 9.1. ^1^H NMR (Acetone-d_6_, 500 MHz): δ 8.58 (s, 1H), 7.68–7.67 (m, 1H), 7.62–7.60 (m, 5H), 7.37–7.28 (m, 7H), 6.87 (ddd, 1H, *J* = 8.1, 2.5, 0.9 Hz) ppm. ^13^C­{^1^H} NMR (Acetone-d_6_, 125 MHz): δ 158.7, 146.8, 133.0, 130.6, 129.2, 128.8, 128.0, 117.4, 116.3, 113.2 ppm.

##### 4.1.2.4 4-(4,5-Diphenylimidazol-2-yl)­phenol (L3)

3.55 g (93%), colorless amorphous solid, *R*_f_ = 0.51 (SiO_2_, Hex/EtOAc 1:1). Anal. Calcd for C_21_H_16_N_2_O: C, 80.7; H, 5.2; N, 9.0; O, 5.1. Found: C, 80.4; H, 5.3; N, 8.8. ^1^H NMR (Acetone-d_6_, 500 MHz): δ 8.00–7.99 (m, 2H), 7.61–7.59 (m, 4H), 7.37–7.34 (m, 4H), 7.30–7.27 (m, 2H), 6.95–6.93 (m, 2H) ppm. ^13^C­{^1^H} NMR (DMSO-*d*_6_, 125 MHz): δ 171.1, 158.2, 146.6, 133.5, 128.9, 128.1, 127.5, 127.4, 121.8, 115.9, 79.4, 60.3 ppm.

#### 4.1.3 Protected Lophines P1–3 Preparation

The lophines protected with TBDMS (**P1**–**3**) were prepared according to the methodology previously used by our group,^38^ inspired by Lu et al.^37^ In a 25 mL round-bottom flask, equipped with magnetic stirring 1 mmol (1 equiv) of **L1**–**3**, 5 mmol (5 equiv) of imidazole, 4 mmol (4 equiv) of TBDMSCl, and 10 mL (0.1 mol L^–1^) of dichloromethane (DCM) were added. Such mixture was kept under stirring for 30 min. Then, 10 mL of Hex was added to induce precipitation of the residual salt, the solution was filtered and washed with deionized water five times to completely remove the salt. Magnesium sulfate was added to retain residual water, the solution was filtered, and the solvent was removed by rotary evaporation and under vacuum (10^–3^ mbar, 30 min). The protected lophines **P1**–**3** were purified by flash chromatographic column (SiO_2_, Hex/EtOAc = 4:1) and the obtained colorless crystals were dried under vacuum for 3 h. For derivative **P1** a recrystallization with Hex after the chromatographic column was needed to achieve the desired degree of purity. **P1**–**3** were characterized by NMR (^1^H and ^13^C, Figures S8–S10) and EA.

##### 4.1.3.1 2-(2-((*tert*-Butyldimethylsilyl)­oxy)­phenyl)-4,5-diphenylimidazole (P1)

0.23 g (42%), colorless crystalline solid, *R*_f_ = 0.74 (SiO_2_, Hex/EtOAc 4:1). Anal. Calcd for C_27_H_30_N_2_OSi: C, 76.0; H, 7.1; N, 6.6; O, 3.7; Si, 6.6. Found: C, 76.3; H, 7.2; N, 6.9. ^1^H NMR (CDCl_3_, 500 MHz): δ 8.42 (dd, 1H, *J* = 7.8, 1.8 Hz), 7.65–7.48 (m, 5H), 7.36–7.29 (m, 6H), 7.24 (ddd, 1H, *J* = 8.2, 7.3, 1.8 Hz), 7.10 (ddd, 1H, *J* = 15.7, 7.9, 1.1 Hz), 6.96 (dd, 1H, *J* = 8.2, 0.9 Hz), 0.98 (s, 9H), 0.34 (s, 6H) ppm. ^13^C­{^1^H} NMR (CDCl_3_, 125 MHz): δ 152.2, 144.4, 129.4, 129.3, 128.7, 127.9, 122.3, 120.5, 119.4, 29.8, 26.1, −3.6 ppm.

##### 4.1.3.2 2-(3-((*tert*-Butyldimethylsilyl)­oxy)­phenyl)-4,5-diphenylimidazole (P2)

0.43 g (79%), colorless crystalline solid, *R*_f_ = 0.73 (SiO_2_, Hex/EtOAc 4:1). Anal. Calcd for C_27_H_30_N_2_OSi: C, 76.0; H, 7.1; N, 6.6; O, 3.7; Si, 6.6. Found: C, 76.2; H, 7.0; N, 7.0. ^1^H NMR (CDCl_3_, 500 MHz): δ 7.56–7.54 (m, 4H), 7.50 (ddd, 1H, *J* = 7.7, 1.6, 0.9 Hz), 7.43–7.42 (m, 1H), 7.35–7.27 (m, 7H), 6.86 (ddd, 1H, *J* = 8.1, 2.4, 0.9 Hz), 1.01 (s, 9H), 0.23 (s, 6H) ppm. ^13^C­{^1^H} NMR (CDCl_3_, 125 MHz): δ 156.3, 146.0, 132.8, 131.3, 130.1, 128.7, 128.0, 127.6, 120.7, 118.6, 117.4, 29.8, 25.8, −4.4 ppm.

##### 4.1.3.3 2-(4-((*tert*-Butyldimethylsilyl)­oxy)­phenyl)-4,5-diphenylimidazole (P3)

0.37 g (67%), colorless crystalline solid, *R*_*f*_ = 0.57 (SiO_2_, Hex/EtOAc 4:1). Anal. Calcd for C_27_H_30_N_2_OSi: C, 76.0; H, 7.1; N, 6.6; O, 3.7; Si, 6.6. Found: C, 75.7; H, 7.4; N, 6.8. ^1^H NMR (CDCl_3_, 500 MHz): δ 7.77 (d, 2H, J = 8.6 Hz), 7.54 (s, 4H), 7.34–7.28 (m, 6H), 6.90 (d, 2H, *J* = 8.6 Hz), 1.01 (s, 9H), 0.23 (s, 6H) ppm. ^13^C­{^1^H} NMR (CDCl_3_, 125 MHz): δ 156.7, 146.3, 128.7, 127.9, 127.4, 126.9, 123.5, 120.7, 25.8, 18.4, −4.2 ppm.

#### 4.1.4 Hydroperoxides H1–3 Preparation

Hydroperoxides **H1**–**3** were prepared according to the methodology previously used by our group,^38^ inspired by Lu et al.^37^ A 100 mL Schlenk reaction tube was charged with 45 mL (ca. 13.6 mmol L^–1^) of an 8:1 DCM/methanol mixture and 0.61 mmol (1 equiv) of **P1**–**3**. A catalytic amount of methylene blue was added, the mixture was kept under magnetic stirring, and molecular oxygen was bubbled using a glass tube with a fritted glass tip. The solution was irradiated under a 665 nm LED array while the temperature was maintained at 4 °C using a water-ice bath. The singlet oxygen produced through photosensitization reacted with the imidazolyl moiety of **P1**–**3** by an ene reaction, producing hydroperoxides **H1**–**3**. The reaction was monitored using thin-layer chromatography (TLC), and the presence of organic peroxide was indicated by a potassium iodide solution in aqueous acetic acid. After completion, the methanol was removed by rotary evaporation, and the resulting reaction mixture was filtered through a 2.5 × 10 cm silica gel layer and eluted with DCM to remove methylene blue. **H1**–**3** were purified by recrystallization with EtOAc/Hex, without heating to avoid their decomposition. These were filtered under vacuum, washed with cold Hex (2 × 50 mL), and the obtained colorless crystals were dried under vacuum for 3 h. **H1**–**3** were characterized by NMR (^1^H and ^13^C, Figures S11–S13) and EA.

##### 4.1.4.1 2-(2-((*tert*-Butyldimethylsilyl)­oxy)­phenyl)-4-hydroperoxy-4,5-diphenylimidazole (H1)

0.11 g (54%), pale yellow crystalline solid, *R*_f_ = 0.42 (SiO_2_, Hex/EtOAc 4:1). Anal. Calcd for C_27_H_30_N_2_O_3_Si: C, 70.7; H, 6.6; N, 6.1; O, 10.5; Si, 6.1. Found: C, 70.8; H, 6.8; N, 6.2. ^1^H NMR (CDCl_3_, 500 MHz): δ 8.32–8.30 (m, 2H), 7.47–7.44 (m, 5H), 7.34–7.30 (m, 5H), 7.13–7.10 (m, 1H), 6.88 (d, 1H, *J* = 8.1 Hz), 2.06 (s, 1H), 0.79 (s, 9H), 0.02 (d, 6H, *J* = 9.7 Hz) ppm. ^13^C­{^1^H} NMR (CDCl_3_, 125 MHz): δ 172.2, 155.7, 152.1, 144.3, 134.8, 132.6, 132.4, 130.2, 128.6, 127.9, 127.1, 122.2, 121.1, 120.8, 119.4, 115.5, 29.8, 26.1, 25.8, −3.7 ppm.

##### 4.1.4.2 2-(3-((*tert*-Butyldimethylsilyl)­oxy)­phenyl)-4-hydroperoxy-4,5-diphenylimidazole (H2)

0.13 g (59%), pale yellow crystalline solid, *R*_f_ = 0.44 (SiO_2_, Hex/EtOAc 4:1). Anal. Calcd for C_27_H_30_N_2_O_3_Si: C, 70.7; H, 6.6; N, 6.1; O, 10.5; Si, 6.1. Found: C, 70.7; H, 6.7; N, 6.3. ^1^H NMR (CDCl_3_, 500 MHz): δ 8.39–8.37 (m, 2H), 7.64–7.62 (m, 1H), 7.60–7.57 (m, 1H), 7.52–7.49 (m, 4H), 7.43–7.42 (m, 1H), 7.34–7.31 (m, 3H), 7.10 (t, 1H, *J* = 7.9 Hz), 6.84 (ddd, 1H, *J* = 8.1, 2.5, 1.0 Hz), 1.60 (s, 1H), 0.94 (s, 9H), 0.11 (d, 6H, *J* = 8.3 Hz) ppm. ^13^C­{^1^H} NMR (CDCl_3_, 125 MHz): δ 191.3, 172.4, 155.7, 134.8, 133.2, 131.5, 130.9, 130.4, 129.5, 128.9, 128.8, 126.8, 124.4, 123.4, 121.1, 115.5, 29.9, 25.8, – 4.4 ppm.

##### 4.1.4.3 2-(4-((*tert*-Butyldimethylsilyl)­oxy)­phenyl)-4-hydroperoxy-4,5-diphenylimidazole (H3)

0.22 g (62%), pale yellow crystalline solid, *R*_f_ = 0.36 (SiO_2_, Hex/EtOAc 4:1). Anal. Calcd for C_27_H_30_N_2_O_3_Si: C, 70.7; H, 6.6; N, 6.1; O, 10.5; Si, 6.1. Found: C, 71.0; H, 6.7; N, 5.9. ^1^H NMR (CDCl_3_, 500 MHz): δ 8.39–8.37 (m, 2H), 7.89–7.86 (m, 2H), 7.60–7.57 (m, 1H), 7.53–7.47 (m, 4H), 7.33–7.30 (m, 3H), 6.69–6.66 (m, 2H), 1.60 (s, 1H), 1.01 (s, 9H), 0.23 (d, 6H, *J* = 5.1 Hz) ppm. ^13^C­{^1^H} NMR (CDCl_3_, 125 MHz): δ 190.8, 172.0, 159.7, 135.1, 133.1, 132.1, 130.9, 130.3, 129.4, 128.9, 128.8, 126.7, 123.3, 120.1, 115.4, 25.8, 18.4, −4.2, −4.2 ppm.

#### 4.1.5 Silylperoxides S1–3 Preparation

Silylperoxides **S1**–**3** were prepared according to the methodology previously used by our group,^38^ inspired by Lu et al.^37^ A 25 mL round-bottom flask was charged with 10 mL (0.092 mol L^–1^) of DCM, 0.92 mmol (1 equiv) of **H1**–**3**, 4.6 mmol (5 equiv) of imidazole, and 3.7 mmol (4 equiv) of TBDMSCl. The mixture was kept under vigorous magnetic stirring at room temperature for 1 h. The reaction was followed by TLC (20:1 Hex/EtOAc), and the presence of silylperoxide was assessed with a 1.0 mol L^–1^ TBAF solution in THF, which produced a rapid light flash at the corresponding TLC spot, visible to the naked eye. Upon reaction completion, the solution was filtered and DCM was removed by rotary evaporation. The obtained solid was solubilized with Hex and washed with an equal volume of cold deionized water (5 × ) to remove residual salt. Magnesium sulfate was added to the organic layer, the mixture was filtered, and the solvent was removed by rotary evaporation and dried under vacuum (10^–3^ mbar, 30 min). Silylperoxides **S1**–**3** were purified by flash chromatography column (SiO_2_, Hex/EtOAc 9:1). The obtained colorless crystals were dried under vacuum for 3 h and were characterized by NMR (^1^H and ^13^C, Figures S14–S16) and EA.

##### 4.1.5.1 2-(2-((*tert*-Butyldimethylsilyl)­oxy)­phenyl)-4-((*tert*-butyldimethylsilyl)­peroxy)-4,5-diphenylimidazole (S1)

0.20 g (70%), pale yellow crystalline solid, *R*_f_ = 0.61 (SiO_2_, Hex/EtOAc 9:1). Anal. Calcd for C_33_H_44_N_2_O_3_Si_2_: C, 69.2; H, 7.7; N, 4.9; O, 8.4; Si, 9.8. Found: C, 69.5; H, 7.8; N, 4.9. ^1^H NMR (CDCl_3_, 500 MHz): δ 8.23–8.20 (m, 2H), 8.06 (dd, 1H, *J* = 7.7, 1.8 Hz), 7.52–7.49 (m, 1H), 7.43–7.40 (m, 2H), 7.37–7.34 (m, 3H), 7.31–7.28 (m, 3H), 7.06–7.03 (m, 1H), 6.96 (dd, 1H, J = 8.2, 0.7 Hz), 0.92 (s, 9H), 0.87 (s, 9H), 0.20 (d, 6H, *J* = 6.4 Hz), 0.16 (d, 6H, *J* = 1.6 Hz) ppm. ^13^C­{^1^H} NMR (CDCl_3_, 125 MHz): δ 189.8, 171.7, 155.7, 135.1, 132.6, 132.3, 132.2, 131.1, 130.0, 129.2, 128.6, 128.4, 126.8, 124.4, 121.1, 120.7, 114.9, 26.2, 26.0, 18.5, 18.5, −3.9, −4.1, −5.3, −5.3 ppm.

##### 4.1.5.2 2-(3-((*tert*-Butyldimethylsilyl)­oxy)­phenyl)-4-((*tert*-butyldimethylsilyl)­peroxy)-4,5-diphenylimidazole (S2)

0.13 g (65%), pale yellow crystalline solid, *R*_f_ = 0.62 (SiO_2_, Hex/EtOAc 9:1). Anal. Calcd for C_33_H_44_N_2_O_3_Si_2_: C, 69.2; H, 7.7; N, 4.9; O, 8.4; Si, 9.8. Found: C, 69.5; H, 7.4; N, 4.7. ^1^H NMR (CDCl_3_, 500 MHz): δ 8.24–8.21 (m, 2H), 8.09–8.07 (m, 1H), 7.95–7.94 (m, 1H), 7.53–7.49 (m, 1H), 7.45–7.42 (m, 2H), 7.39–7.36 (m, 1H), 7.34–7.31 (m, 2H), 7.29–7.27 (m, 3H), 7.04 (ddd, 1H, *J* = 8.1, 2.6, 1.0 Hz), 1.02 (s, 9H), 0.85 (s, 9H), 0.26 (d, 6H, *J* = 2.6 Hz), 0.21 (s, 3H), 0.16 (s, 3H) ppm. ^13^C­{^1^H} NMR (CDCl_3_, 125 MHz): δ 191.2, 171.5, 156.0, 134.8, 133.1, 132.5, 131.2, 129.8, 129.6, 129.2, 128.8, 128.6, 126.2, 123.9, 123.3, 121.3, 115.2, 29.9, 26.2, 25.9, 18.5, 18.4, −4.2, −4.2, −5.3, −5.3 ppm.

##### 4.1.5.3 2-(4-((*tert*-Butyldimethylsilyl)­oxy)­phenyl)-4-((*tert*-butyldimethylsilyl)­peroxy)-4,5-diphenylimidazole (S3)

0.16 g (65%), pale yellow crystalline solid, *R*_f_ = 0.86 (SiO_2_, Hex/EtOAc 9:1). Anal. Calcd for C_33_H_44_N_2_O_3_Si_2_: C, 69.2; H, 7.7; N, 4.9; O, 8.4; Si, 9.8. Found: C, 69.1; H, 7.9; N, 4.5. ^1^H NMR (CDCl_3_, 500 MHz): δ 8.40–8.38 (m, 2H), 8.24–8.22 (m, 2H), 7.52–7.49 (m, 1H), 7.45–7.41 (m, 2H), 7.35–7.33 (m, 2H), 7.29–7.27 (m, 3H), 6.99–6.96 (m, 2H), 1.03 (s, 9H), 0.86 (s, 9H), 0.27 (s, 6H), 0.22 (s, 3H), 0.17 (s, 3H) ppm. ^13^C­{^1^H} NMR (CDCl_3_, 125 MHz): δ 190.9, 171.2, 159.4, 135.2, 132.4, 131.8, 131.2, 129.8, 129.0, 128.8, 128.5, 126.1, 124.9, 120.3, 115.2, 26.2, 25.8, 18.4, 18.4, −4.2, −4.2, −5.3, −5.3 ppm.

#### 4.1.6 Quantum Yields Determination

Values for ϕ_fl_ of **L1**–**3**, **S1**–**3**, and **B1**–**3** were determined in ACN following the relative method^49^ using lophine^45^ as standard for **L1**–**3**, 4-hydroperoxy-2-(4-methoxyphenyl)-4,5-diphenyl-4-imidazole^38^ for **S1**–**3**, and 2,5-diphenyloxazole^75^ for **B1**–**3**. Values for ϕ_cl_ were obtained from the integrated CL intensity-time profiles, using the method reported elsewhere.^3^ Values for ϕ_s_ were determined by numerically dividing ϕ_cl_ by ϕ_fl_, as described elsewhere.^3^

#### 4.1.7 Chemiluminescence Kinetic Assays

The chemiluminescence measurements were performed using an experimental setup and protocol previously validated for related lophine-derived systems,^38^ for which appropriate control experiments and background corrections were established. For CL kinetic assays, fresh stock solutions were prepared for **S2** and **S3** at 1.5 μmol L^–1^ in ACN, for **S1** at 0.15 μmol L^–1^ in ACN, and for TBAF at 1.5 mmol L^–1^ in THF, the latter by diluting a commercial 1.0 mol L^–1^ TBAF solution in THF. Kinetic measurements of light emission were conducted using a Berthold Sirius single tube luminometer, varying the TBAF concentration from 0.037 to 0.210 mmol L^–1^ keeping the silylperoxides concentration constant at 1.27 μmol L^–1^ for **S2** and **S3**, and 0.127 μmol L^–1^ for **S1**. The luminometer test tube was filled with 1.7 mL of silylperoxide stock solution and placed in the measurement chamber of the equipment. Four seconds after the start of data acquisition, two automatic injectors of the luminometer were used to simultaneously add the TBAF stock solution and pure THF. By adjusting the volumes set for both injectors, different TBAF concentrations could be achieved without altering the volume of THF in the luminometer test tube. The total volume added by both injectors was consistently 300 μL to maintain the final volume in the tube at 2.0 mL, ensuring the concentration of silylperoxides remained constant. Kinetic assays were performed in three replicas for each TBAF concentration to report mean values and standard deviations for all rate constants.

### 4.2 DFT and TD-DFT Calculations

The Coulomb-attenuated hybrid exchange-correlation functional (CAM-B3LYP)^76^ was employed in combination with the cc-pVTZ basis set and implicit solvent model using the conductor-like polarizable continuum model (CPCM) in acetonitrile within the Orca (version 5.0.4, unless otherwise noted) program in all calculations. Additionally, we use the def2/J auxiliary basis, and the RIJCOSX approximation was employed to expedite the calculation. While the stationary points, i.e., **D1**-**3** and **B1**-**3** ^–^ reaction product, on S_0_ potential energy surface was optimized applying restricted Kohn–Sham Density Functional Theory theory (RKS-DFT) formalism, the transition states was calculated using broken-symmetry (BS) with the unrestricted Kohn–Sham Density Functional Theory theory (UKS-DFT). For the intrinsic reaction coordinate (IRC) calculation, we used the BS with the UKS-DFT within the Orca (version 5.0.3) program. We performed vertical excitation TD-DFT calculations on geometries obtained along the S_0_ IRC using an RKS reference without the (BS) formalism.

## Supplementary Material





## Data Availability

The data underlying this study are available in the published article and its Supporting Information.

## Notes

Optimized geometries of the reactants (**D1**–**D3**), transition states (**TS1**–**TS3**), and reaction products (**B1**–**B3** ^–^) were collected in an .xyz file, together with their respective electronic energies and the number of imaginary frequencies associated with each optimized structure (XYZ)
Spectrophotometric and chemiluminescence spectra, kinetic data, NMR spectra, and quantum chemistry calculations (PDF)
