# Supplementary material for: Regioisomeric Control of Chemiexcitation Efficiency in Lophine Silylperoxides through Reaction-Path Topology and Excited-State Accessibility
Source: ACS Org Inorg Au. 2026 Jun 29;6(4):460–73. doi: 10.1021/acsorginorgau.6c00037 (PMC13449424; doi:10.1021/acsorginorgau.6c00037)
Supplement: Supplementary file 1 [file gg6c00037_si_002.pdf]

# Supporting Information

*for*

# Regioisomeric Control of Chemiexcitation Efficiency in Lophine Silylperoxides through Reaction-Path Topology and Excited-State Accessibility

Diego Ulysses Melo,[†,‡] Andreia Boaro,[†,‡] Roberta Albino dos Reis,[†] Leonardo Martins Carneiro,[†] Paula Homem-de-Mello,[†] and Fernando Heering Bartoloni[*,†]

†*Centro de Ciências Naturais e Humanas, Universidade Federal do ABC, Santo André, São Paulo, Brazil, 09210-580*

‡*D.U.M. and A.B. contributed equally to this work*

E-mail: fernando.bartoloni@ufabc.edu.br

**List of Contents**

| | Page |
|---|---|
| **1. Spectrophotometric Data** | S2 |
| **2. Quantum Chemistry Calculation** | S6 |
| **3. NMR Spectra** | S8 |

# 1. Spectrophotometric Data

Table S1: Values of maximum emission intensity ($I_{max}$), rate constants of decay ($k^1_{obs}$), and increase in emission intensity ($k^2_{obs}$) for the kinetic assays of variation of TBAF concentration for **S1**.

| [TBAF] (mmol $L^{-1}$) | $I_{max}$ (E $s^{-1}$ × $10^{-16}$) | $k^1_{obs}$ ($s^{-1}$) | $k^2_{obs}$ ($s^{-1}$) |
|---|---|---|---|
| 0.037 | 0.46 ± 0.02 | — | 0.076 ± 0.004 |
| 0.045 | 0.97 ± 0.07 | 0.0069 ± 0.0001 | 0.261 ± 0.015 |
| 0.052 | 1.82 ± 0.04 | 0.0254 ± 0.0002 | 0.791 ± 0.040 |
| 0.060 | 2.33 ± 0.05 | 0.0365 ± 0.0005 | 1.088 ± 0.053 |
| 0.067 | 2.92 ± 0.09 | 0.052 ± 0.001 | 1.518 ± 0.004 |
| 0.075 | 3.43 ± 0.17 | 0.064 ± 0.003 | 2.158 ± 0.063 |
| 0.082 | 3.96 ± 0.11 | 0.074 ± 0.002 | 2.803 ± 0.138 |
| 0.090 | 4.74 ± 0.37 | 0.088 ± 0.002 | 4.198 ± 0.183 |
| 0.097 | 5.53 ± 0.61 | 0.097 ± 0.001 | 4.750 ± 0.252 |
| 0.105 | 5.78 ± 0.30 | 0.1104 ± 0.0003 | 5.251 ± 0.025 |
| 0.120 | 8.00 ± 0.83 | 0.135 ± 0.004 | 6.257 ± 0.321 |
| 0.135 | 9.14 ± 0.25 | 0.142 ± 0.001 | 6.910 ± 0.145 |
| 0.150 | 9.93 ± 0.07 | 0.175 ± 0.008 | 7.992 ± 0.424 |
| 0.180 | 10.27 ± 0.01 | 0.211 ± 0.006 | 10.037 ± 0.447 |
| 0.195 | 10.27 ± 0.01 | 0.233 ± 0.008 | 13.025 ± 0.708 |
| 0.210 | 10.24 ± 0.01 | 0.262 ± 0.013 | 17.584 ± 0.568 |

Table S2: Values of maximum emission intensity ($I_{max}$), rate constants of decay ($k^1_{obs}$), and increase in emission intensity ($k^2_{obs}$) for the kinetic assays of variation of TBAF concentration for **S2**.

| [TBAF] (mmol $L^{-1}$) | $I_{max}$ (E $s^{-1}$ × $10^{-16}$) | $k^1_{obs}$ ($s^{-1}$) | $k^2_{obs}$ ($s^{-1}$) |
|---|---|---|---|
| 0.052 | 0.45 ± 0.08 | — | 0.072 ± 0.006 |
| 0.060 | 1.09 ± 0.10 | 0.028 ± 0.002 | 0.218 ± 0.015 |
| 0.067 | 1.82 ± 0.07 | 0.047 ± 0.001 | 0.560 ± 0.037 |
| 0.075 | 2.23 ± 0.14 | 0.060 ± 0.002 | 0.819 ± 0.099 |
| 0.082 | 2.49 ± 0.12 | 0.073 ± 0.001 | 1.142 ± 0.044 |
| 0.090 | 2.86 ± 0.26 | 0.087 ± 0.002 | 1.556 ± 0.009 |
| 0.097 | 3.33 ± 0.27 | 0.108 ± 0.003 | 2.308 ± 0.240 |
| 0.105 | 3.70 ± 0.25 | 0.119 ± 0.001 | 2.273 ± 0.326 |
| 0.120 | 4.25 ± 0.17 | 0.143 ± 0.002 | 3.198 ± 0.157 |
| 0.135 | 5.10 ± 0.25 | 0.173 ± 0.005 | 3.287 ± 0.127 |
| 0.150 | 5.59 ± 0.12 | 0.198 ± 0.004 | 3.660 ± 0.171 |
| 0.180 | 7.05 ± 0.32 | 0.265 ± 0.007 | 3.727 ± 0.061 |
| 0.195 | 7.53 ± 0.27 | 0.294 ± 0.001 | 3.949 ± 0.004 |
| 0.210 | 7.86 ± 0.37 | 0.320 ± 0.009 | 3.466 ± 0.024 |

Table S3: Values of maximum emission intensity ($I_{max}$), rate constants of decay ($k^1_{obs}$), and increase in emission intensity ($k^2_{obs}$) for the kinetic assays of variation of TBAF concentration for **S3**.

| [TBAF] (mmol $L^{-1}$) | $I_{max}$ (E $s^{-1} \times 10^{-16}$) | $k^1_{obs}$ ($s^{-1}$) | $k^2_{obs}$ ($s^{-1}$) |
|---|---|---|---|
| 0.037 | 0.053 ± 0.002 | — | 0.081 ± 0.003 |
| 0.045 | 0.10 ± 0.01 | 0.0219 ± 0.0002 | 0.163 ± 0.024 |
| 0.052 | 0.14 ± 0.02 | 0.0296 ± 0.0002 | 0.251 ± 0.013 |
| 0.060 | 0.35 ± 0.02 | 0.080 ± 0.001 | 1.277 ± 0.047 |
| 0.067 | 0.55 ± 0.01 | 0.110 ± 0.001 | 2.268 ± 0.160 |
| 0.075 | 0.70 ± 0.04 | 0.136 ± 0.001 | 2.939 ± 0.134 |
| 0.082 | 0.82 ± 0.06 | 0.151 ± 0.004 | 2.955 ± 0.102 |
| 0.090 | 1.01 ± 0.05 | 0.163 ± 0.001 | 2.968 ± 0.137 |
| 0.097 | 1.15 ± 0.11 | 0.189 ± 0.007 | 3.359 ± 0.067 |
| 0.105 | 1.18 ± 0.04 | 0.197 ± 0.002 | 3.308 ± 0.080 |
| 0.120 | 1.48 ± 0.06 | 0.232 ± 0.002 | 2.900 ± 0.069 |
| 0.135 | 1.95 ± 0.03 | 0.279 ± 0.008 | 3.267 ± 0.057 |
| 0.150 | 2.35 ± 0.13 | 0.338 ± 0.002 | 3.324 ± 0.136 |
| 0.180 | 3.48 ± 0.08 | 0.410 ± 0.001 | 2.904 ± 0.097 |
| 0.195 | 4.10 ± 0.13 | 0.466 ± 0.002 | 3.153 ± 0.033 |
| 0.210 | 4.93 ± 0.13 | 0.510 ± 0.005 | 2.756 ± 0.027 |

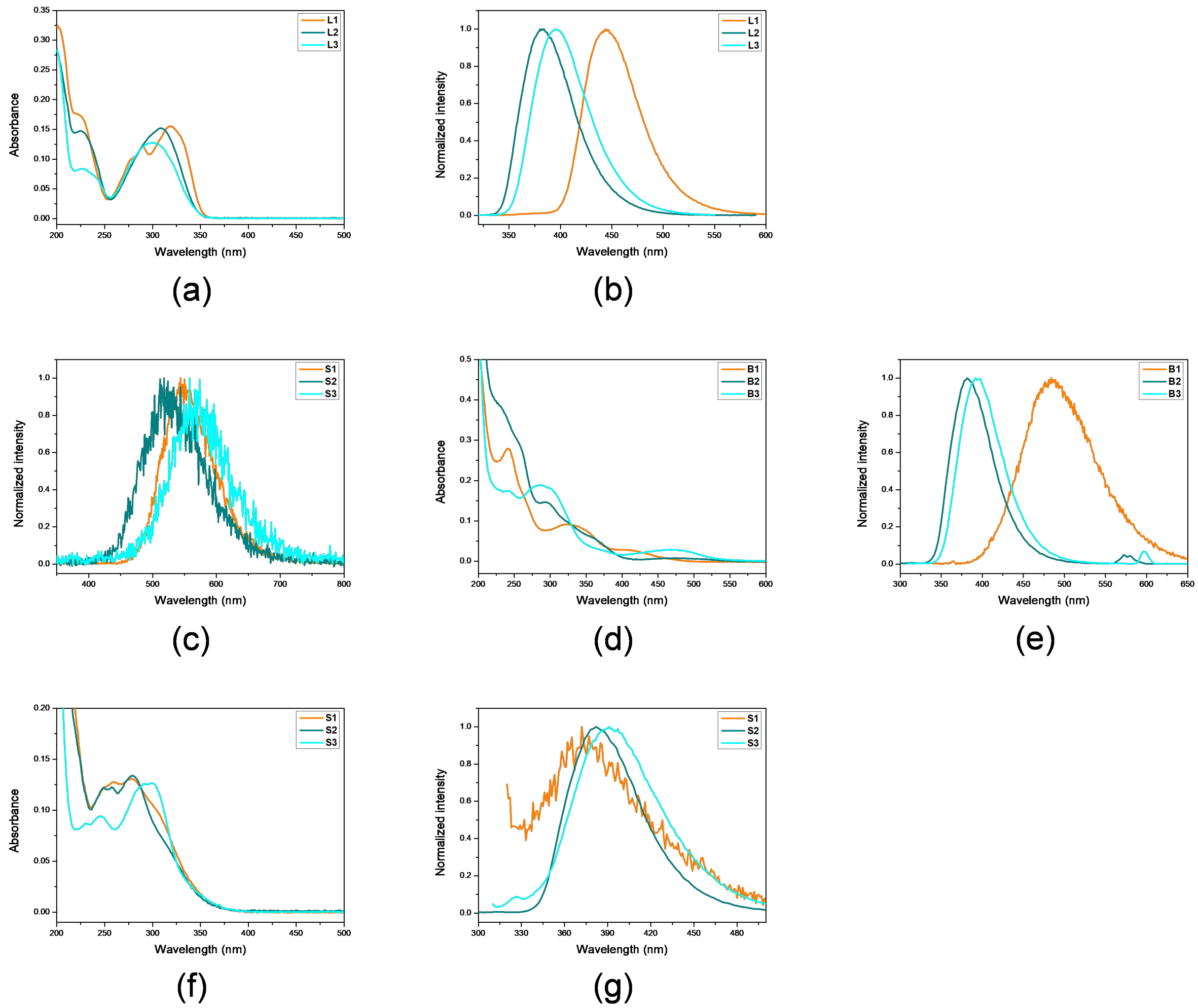


Figure S1: A) and B) Absorption and fluorescence spectra for **L1**–**3**, C) chemiluminescence spectra for **S1**–**3** after addition of tetrabutylammonium fluoride (TBAF), D) and E) absorption and fluorescence spectra for **B1**–**3**, F) and G) absorption and fluorescence spectra for **S1**–**3**.

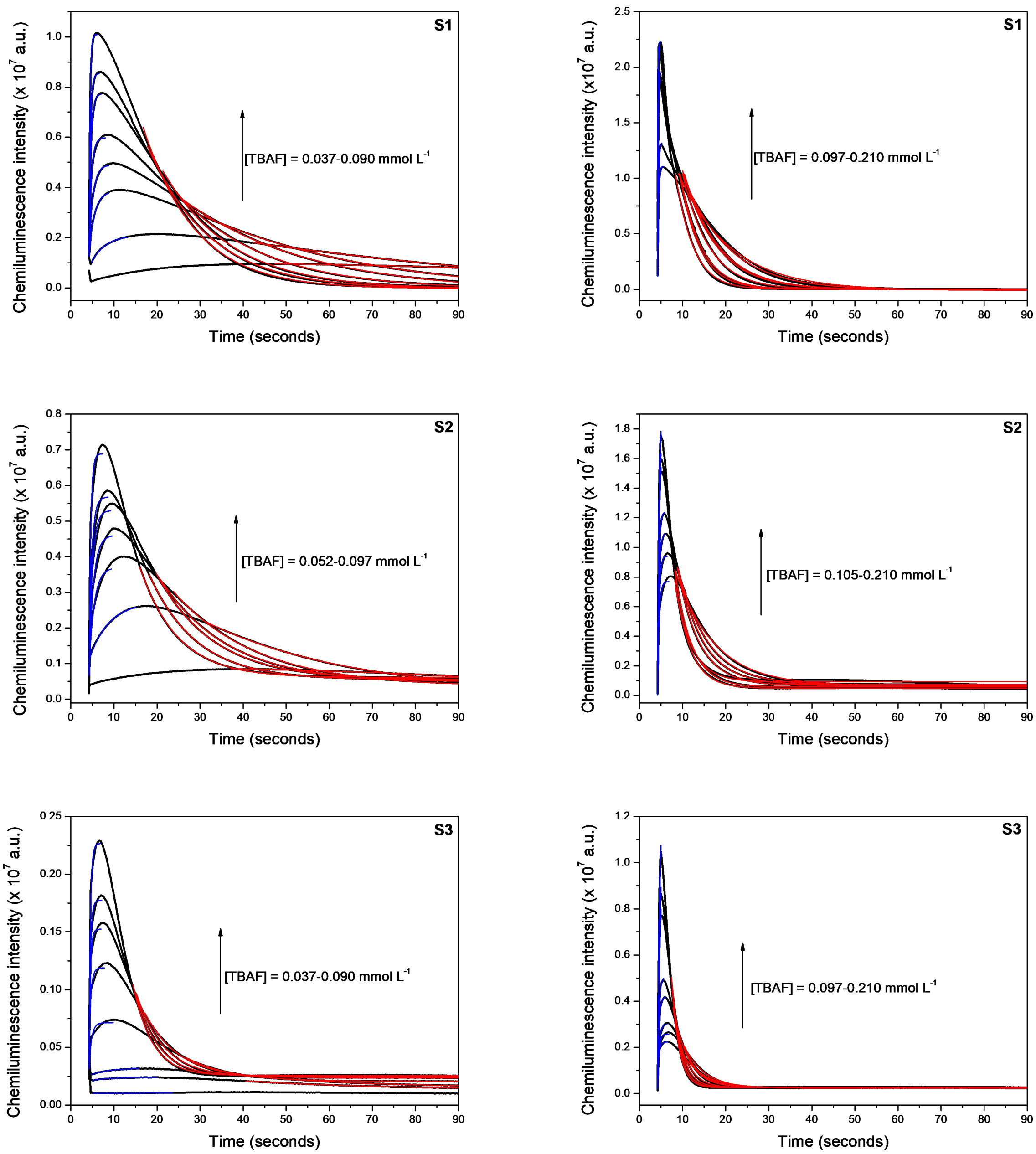


Figure S2: Intensity-time profiles for the kinetic experiments performed in a luminometer for **S1**–**3**. The fall (red line) and rise (blue line) in chemiluminescence intensity of the kinetic profiles (black line) were fitted using equations 2 and 3 to obtain the rate constants $k^1_{\text{obs}}$ and $k^2_{\text{obs}}$, respectively.

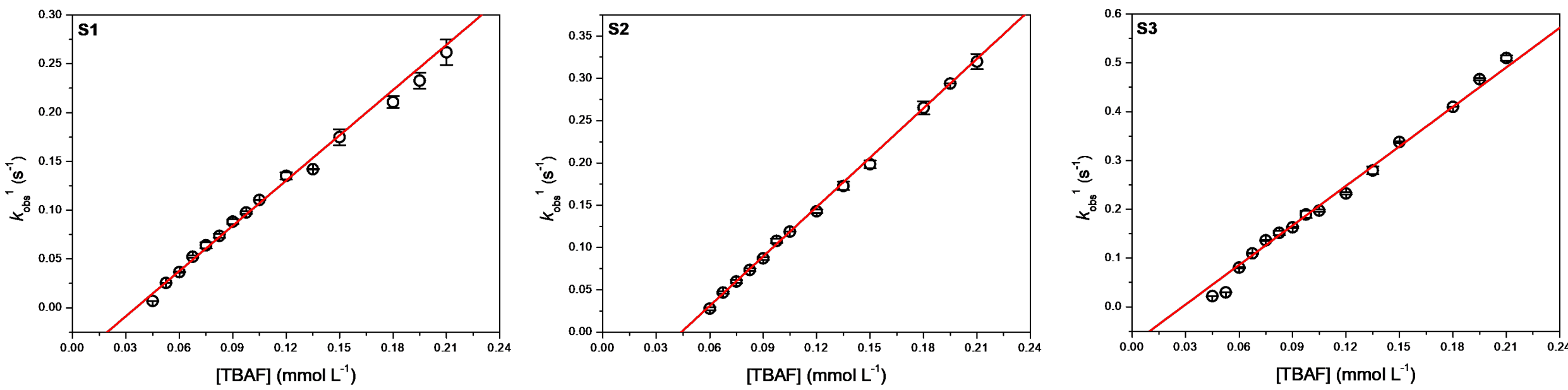


Figure S3: Linear correlation between the observed rate constant of the decay in CL intensity ($k^1_{\text{obs}}$) with the TBAF concentration in the induced decomposition of **S1–3**.

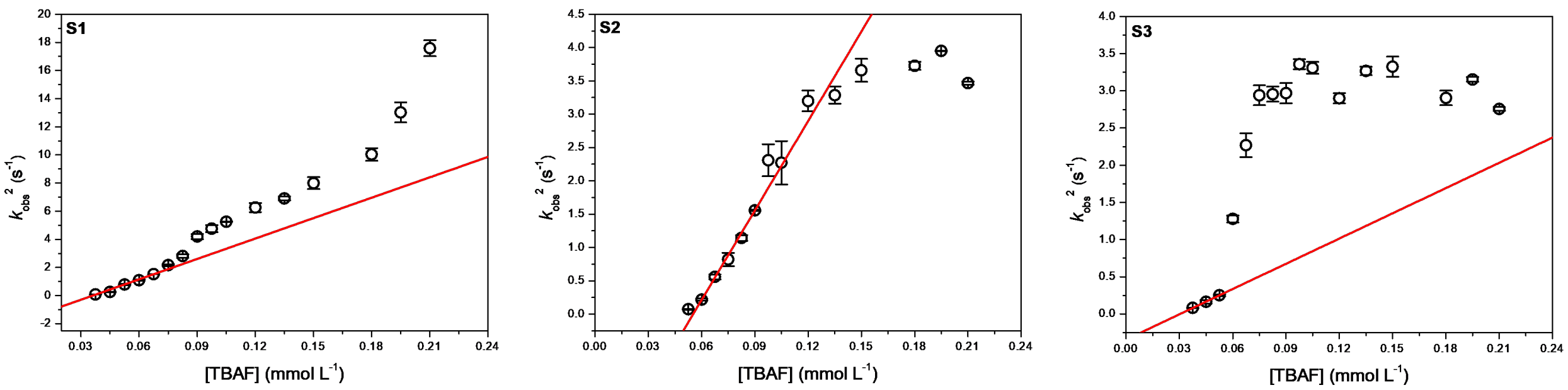


Figure S4: Linear correlation between the observed rate constant of the decay in CL intensity ($k^2_{\text{obs}}$) with the TBAF concentration in the induced decomposition of **S1–3**.

# 2. Quantum Chemistry Calculation

Table S4: Relevant Geometric Parameters of **D1-3**, **TS1-3** and **BS1-3**$^-$ Optimized Geometries at CAM-B3LYP/cc-pVTZ//CPCM(acetonitrile)

| | Bond Length$^a$ | | Dihedral Angle$^b$ |
|---|---|---|---|
| | O-O | C-C | O-C-C-O |
| **D1** | 1.489 | 1.466 | 31.4 |
| **TS1** | 1.756 | 1.497 | 39.1 |
| **B1**$^-$ | 5.689 | 3.950 | 177.3 |
| **D2** | 1.490 | 1.464 | 31.7 |
| **TS2** | 1.745 | 1.512 | 39.3 |
| **B2**$^-$ | 5.383 | 3.832 | −176.5 |
| **D3** | 1.489 | 1.466 | 31.4 |
| **TS3** | 1.755 | 1.498 | 39.6 |
| **B3**$^-$ | 3.237 | 3.024 | 123.6 |

$^a$ Values in Å; $^b$ values in deg.

Table S5: The Population of ChelpG Charges in Highlighted Points at the $S_0$ PEC Calculated by (BS)UCAM-B3LYP//cc-pVTZ//CPCM(acetonitrile) Level of Theory

| Compound | A | TS | B | C | D |
|---|---|---|---|---|---|
| **D1** | −0.855 | −1.230 | −1.854 | −1.415 | −1.455 |
| **D2** | −0.801 | −1.247 | −1.646 | −1.251 | −1.369 |
| **D3** | −0.849 | −1.222 | −1.829 | −1.455 | −1.456 |

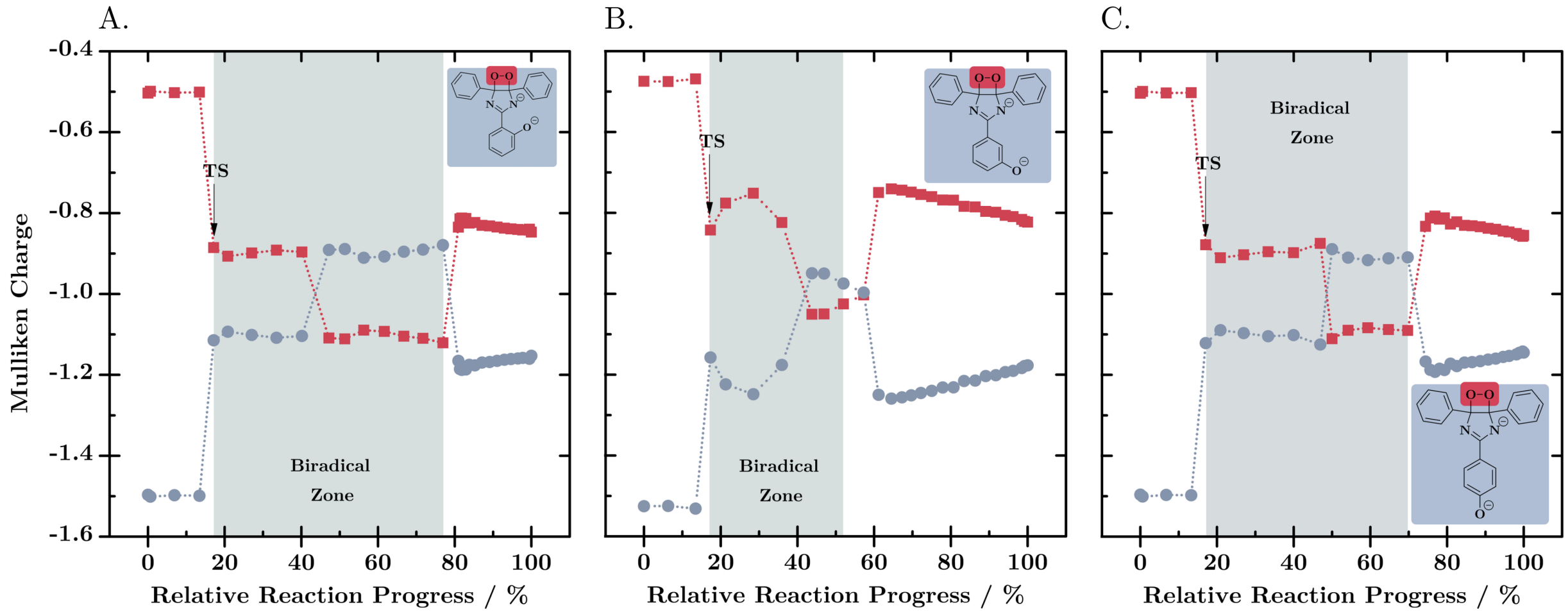


Figure S5: The population of Mulliken charges on oxygens peroxide atoms and rest of the molecule at $S_0$ calculated by (BS)UCAM-B3LYP//cc-pVTZ//CPCM(acetonitrile) level of theory. The relative reaction progress scale was built considering the variation of the dihedral angle across the obtained IRC curve.

## 3. NMR Spectra

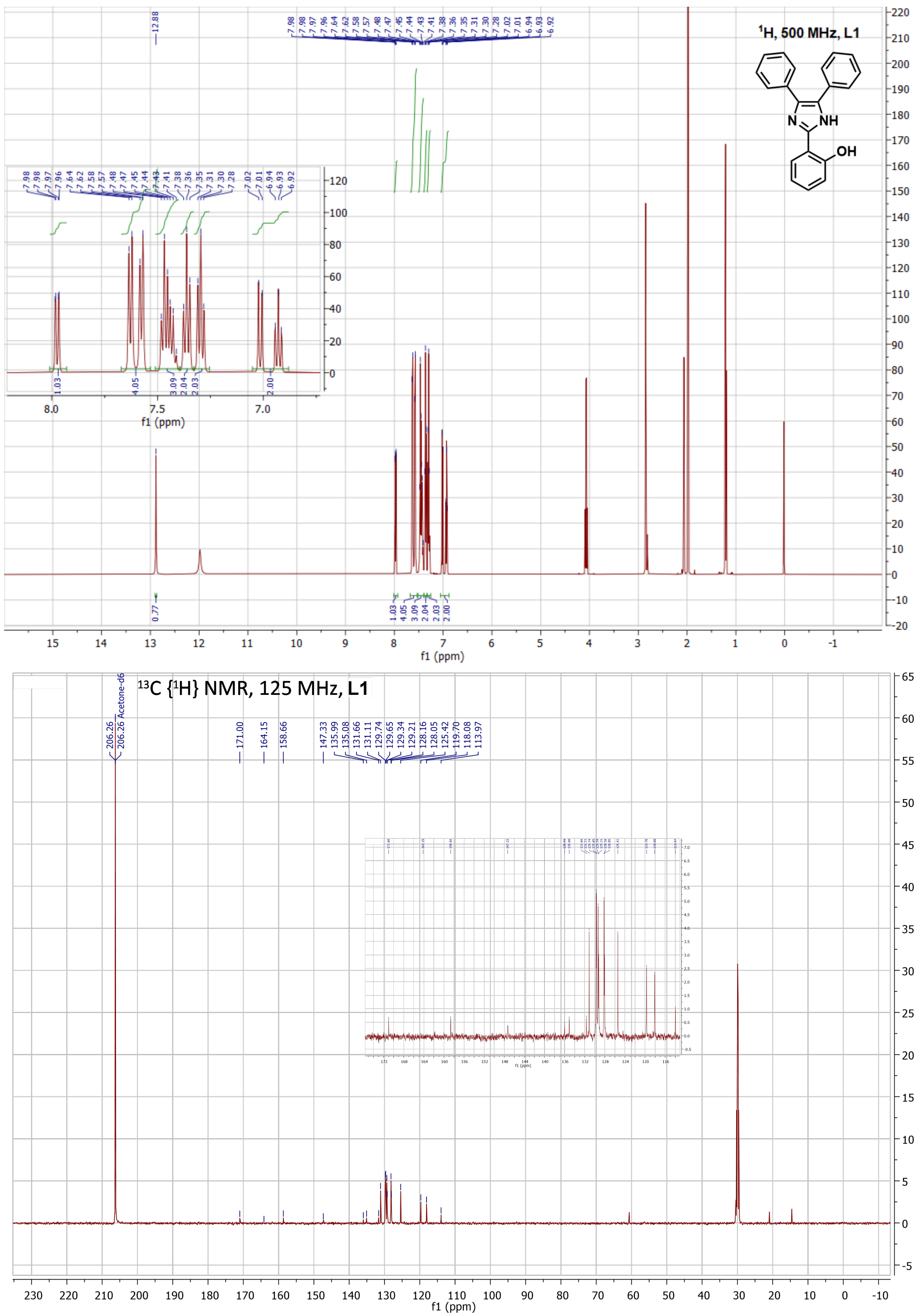


Figure S6: $^{1}$H (top) and $^{13}$C{$^{1}$H} (bottom) NMR spectra for **L1** in acetone-d$_6$.

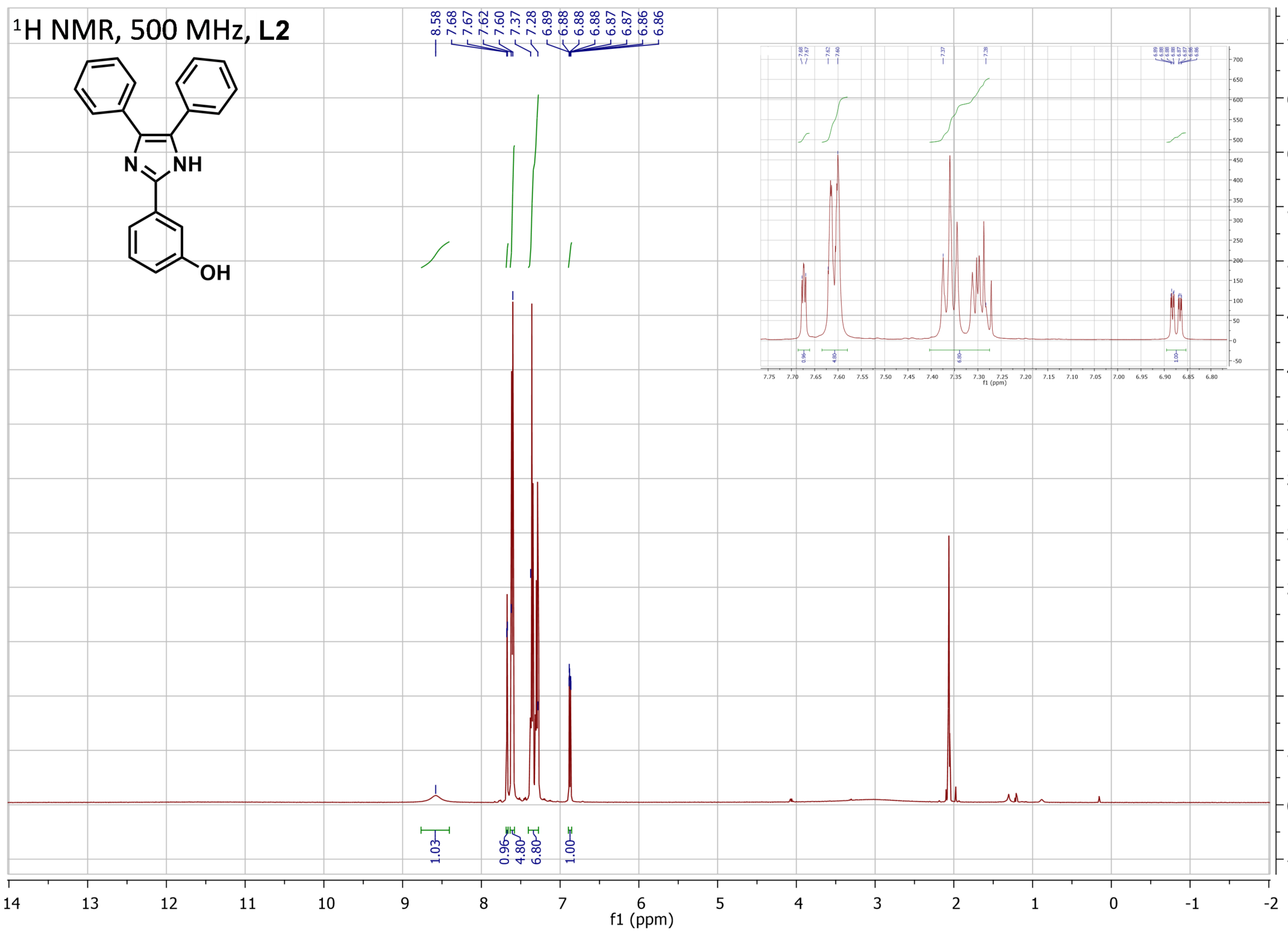


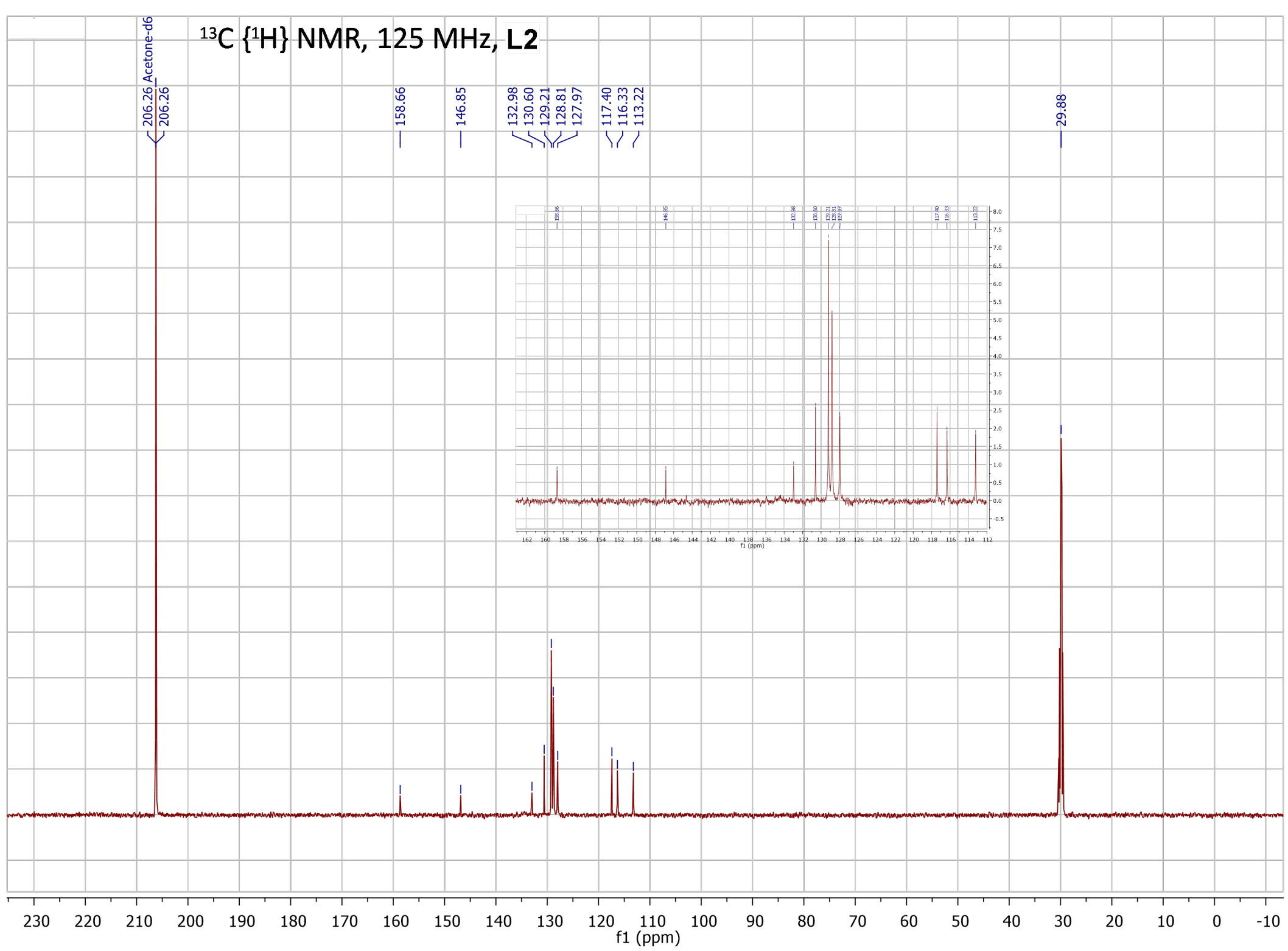


Figure S7: $^1$H (top) and $^{13}$C{$^1$H} (bottom) NMR spectra for **L2** in acetone-$d_6$.

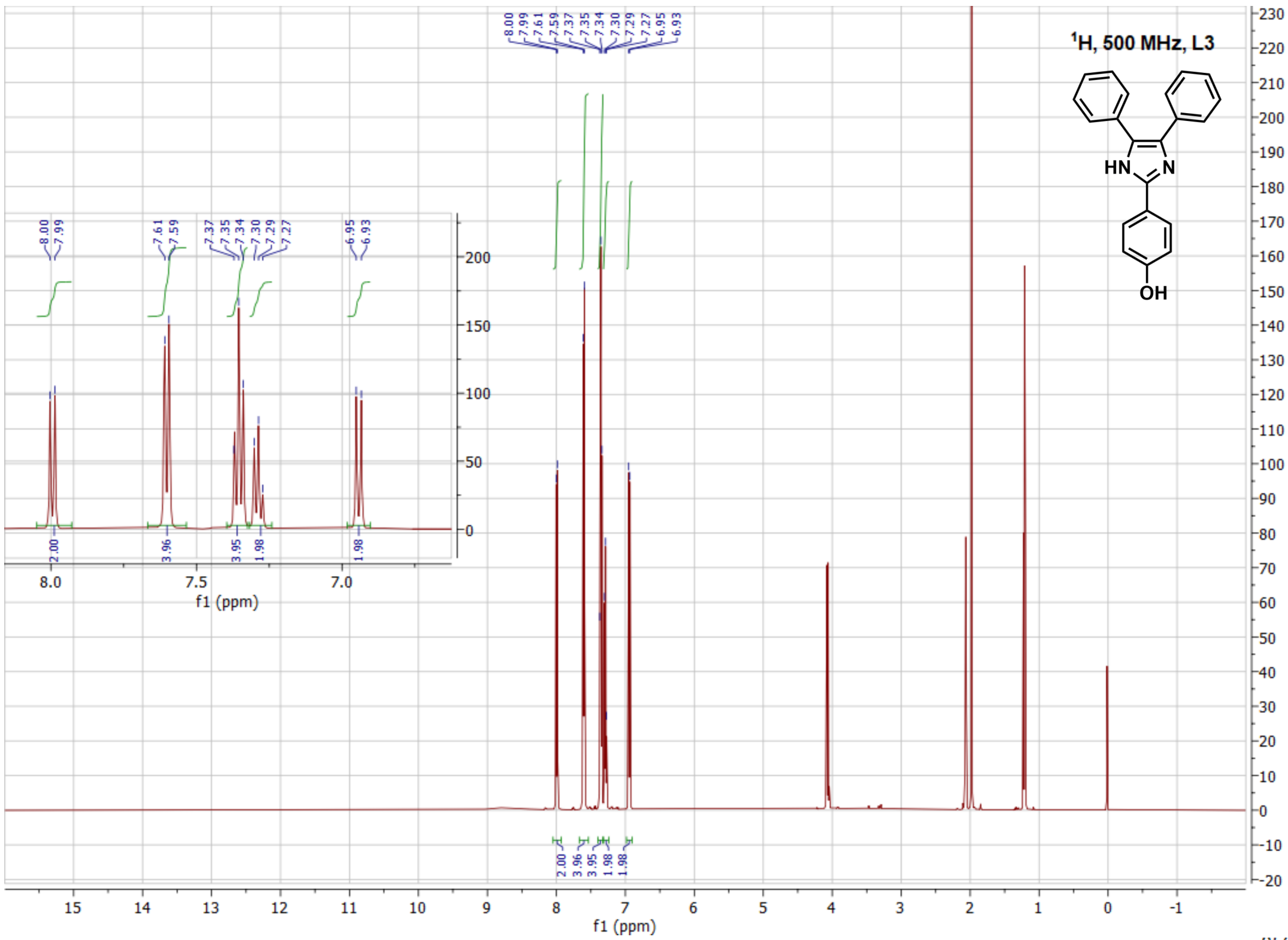


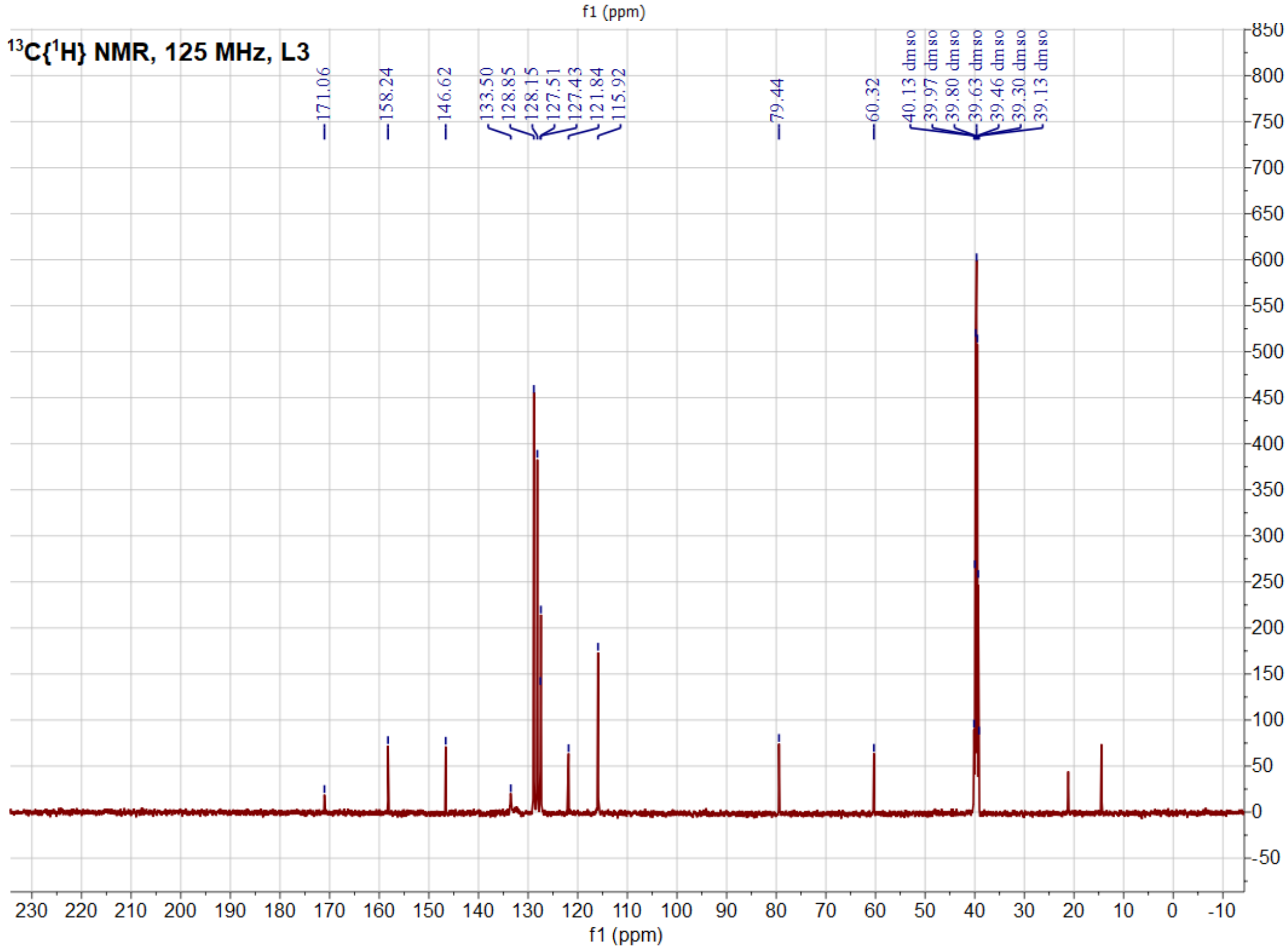


Figure S8: $^1$H (top) and $^{13}$C{$^1$H} (bottom) NMR spectra for **L3** in acetone-$d_6$.

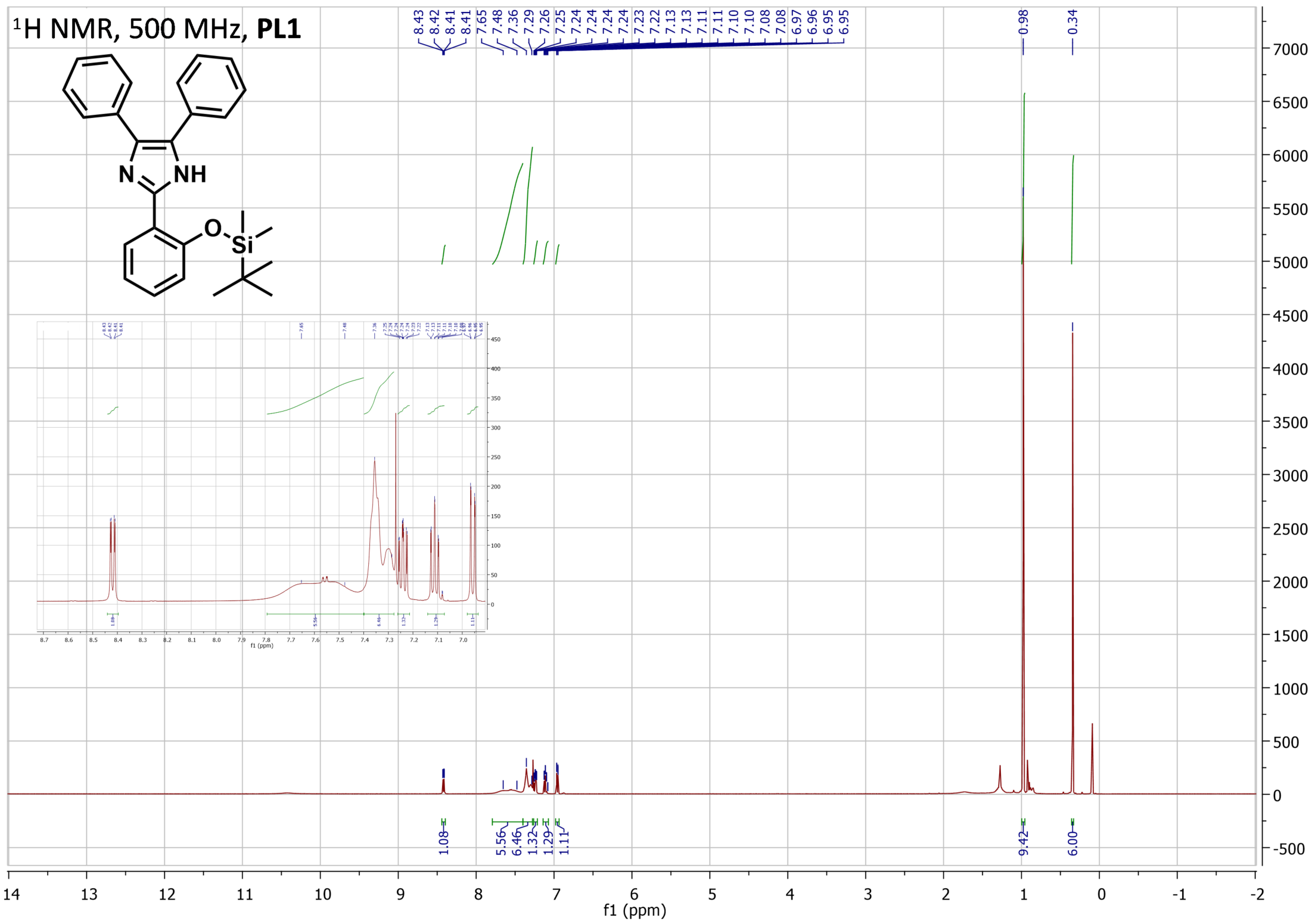


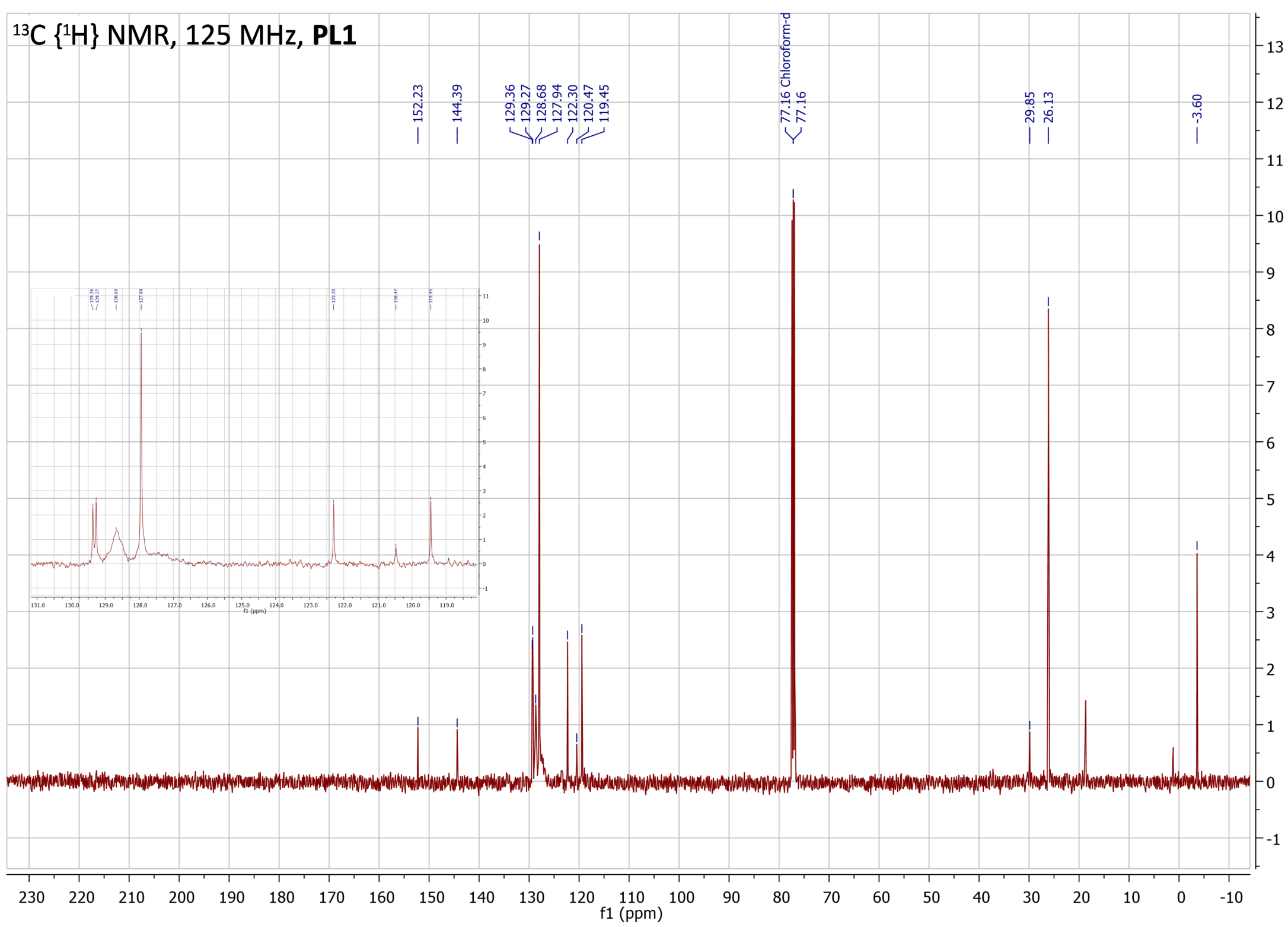


Figure S9: $^{1}$H (top) and $^{13}$C{$^{1}$H} (bottom) NMR spectra for **PL1** in $CDCl_3$.

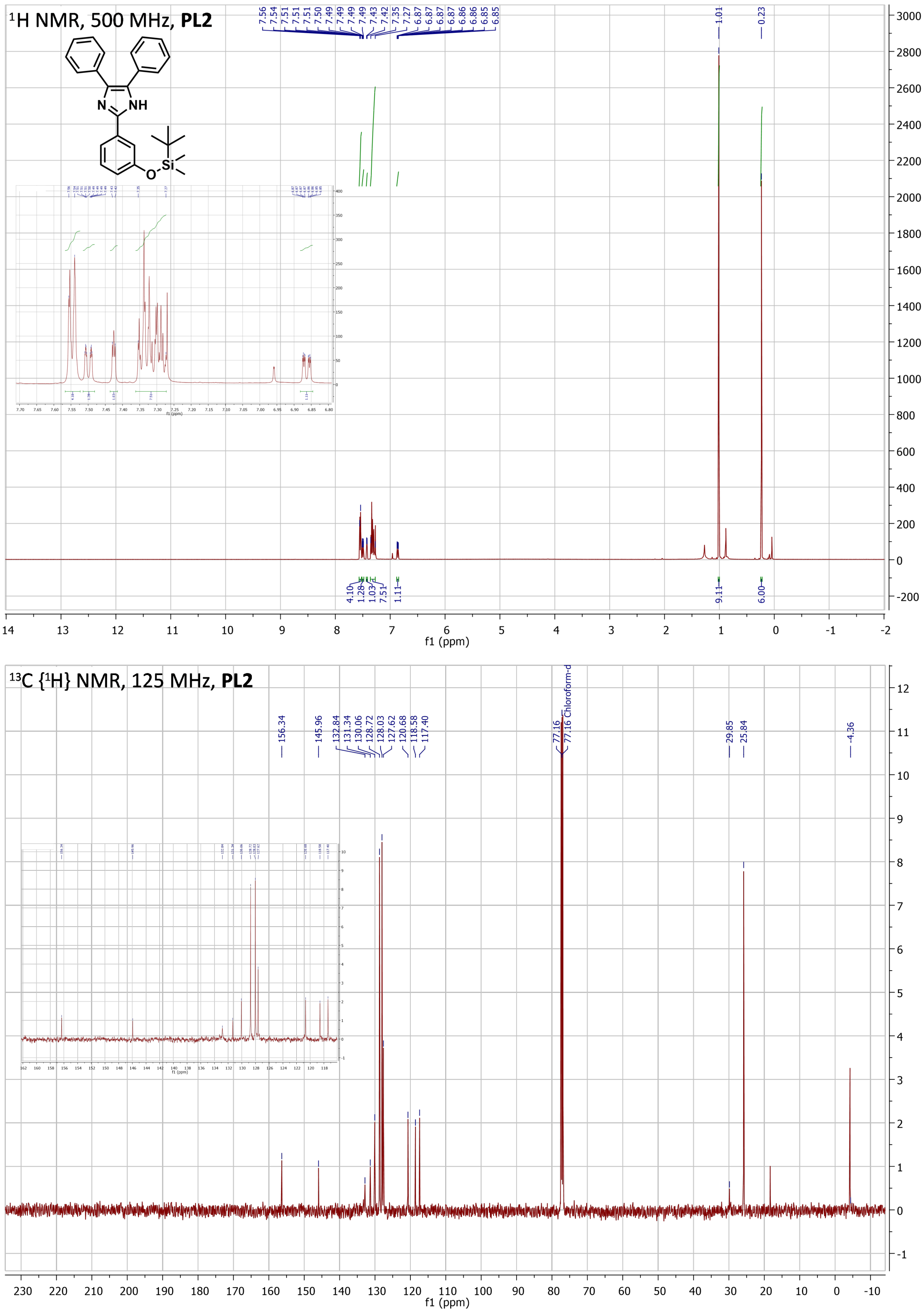


Figure S10: $^1$H (top) and $^{13}$C{$^1$H} (bottom) NMR spectra for **PL2** in $CDCl_3$.

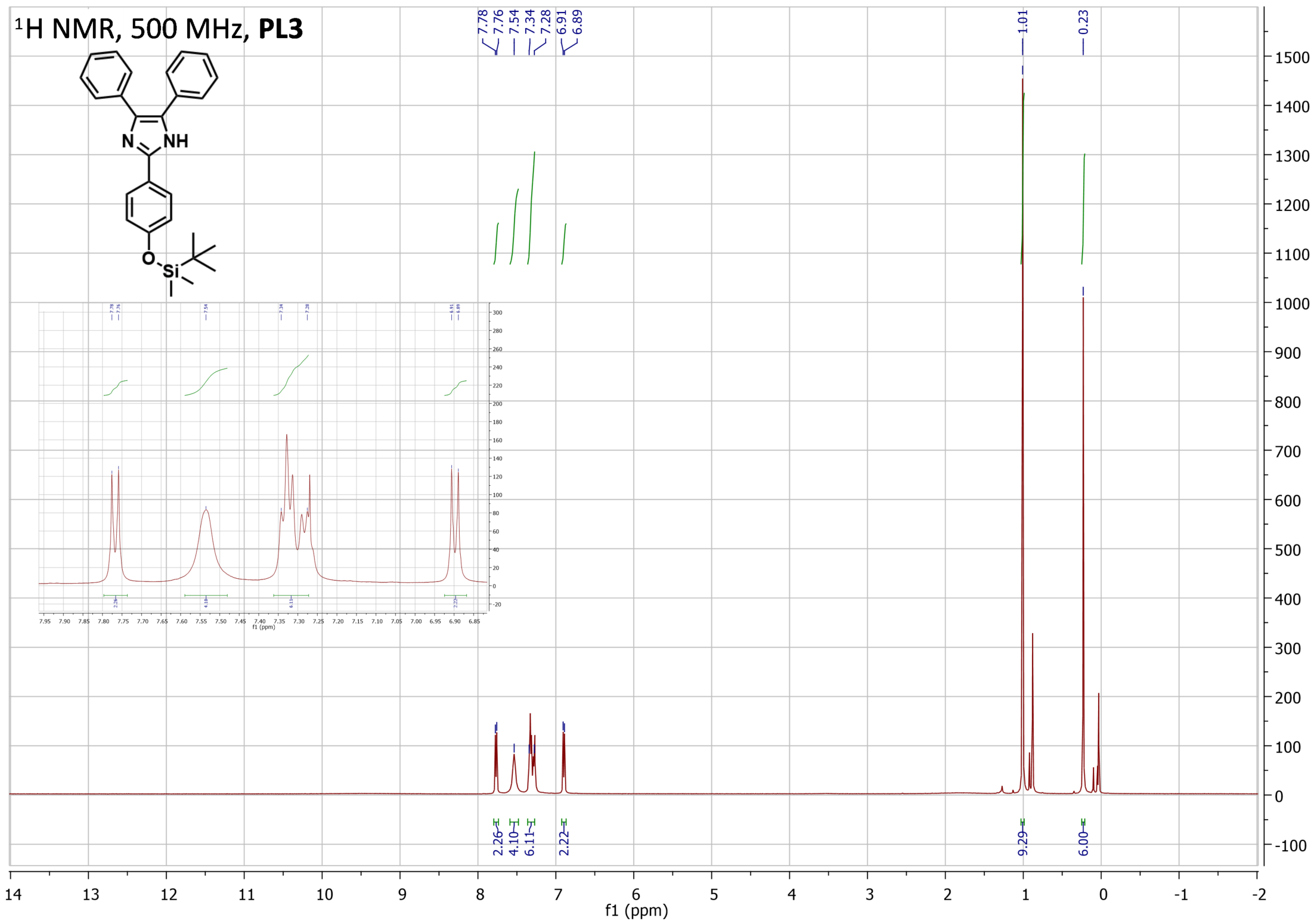


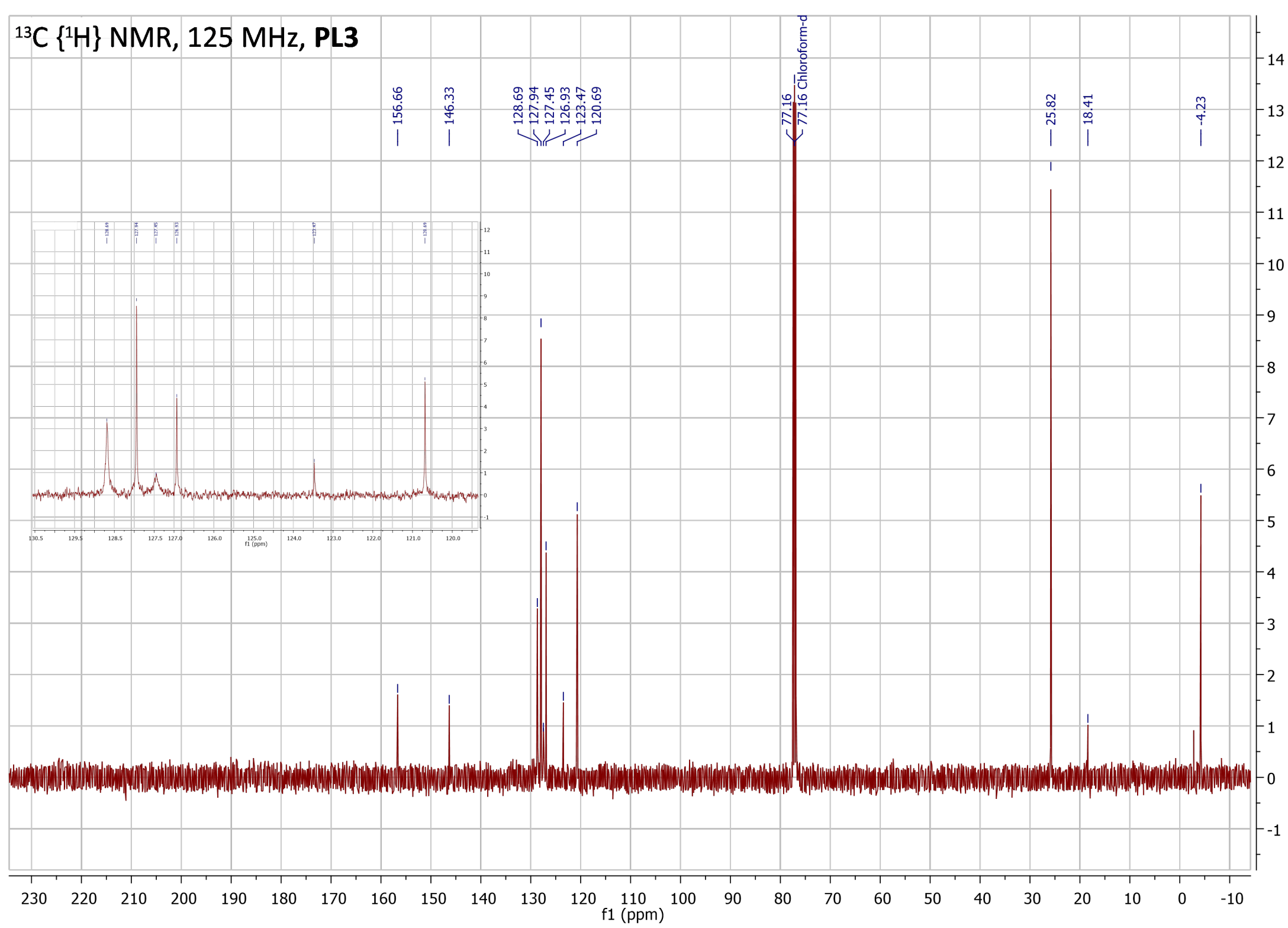


Figure S11: $^{1}$H (top) and $^{13}$C{$^{1}$H} (bottom) NMR spectra for **PL3** in $CDCl_3$.

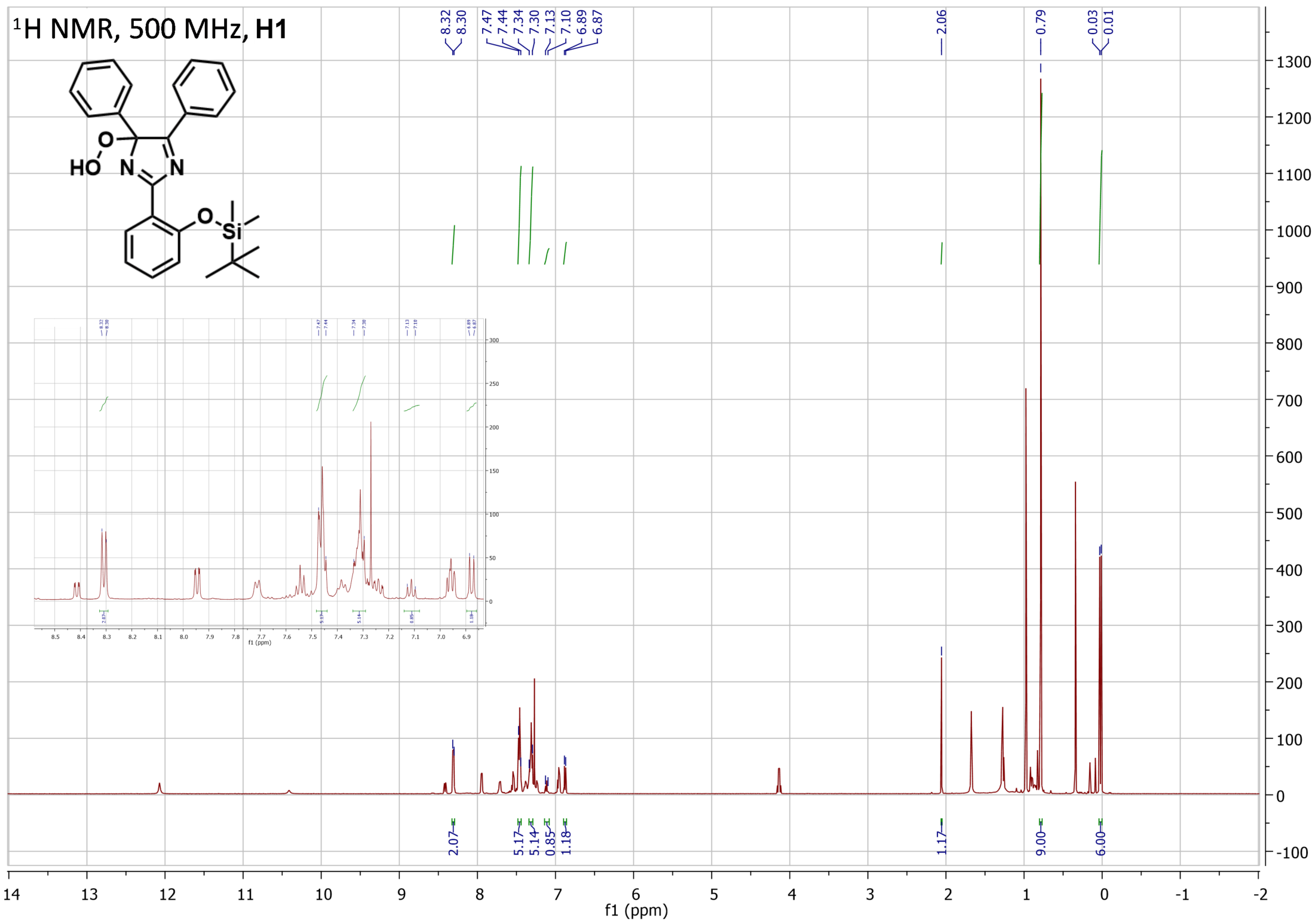

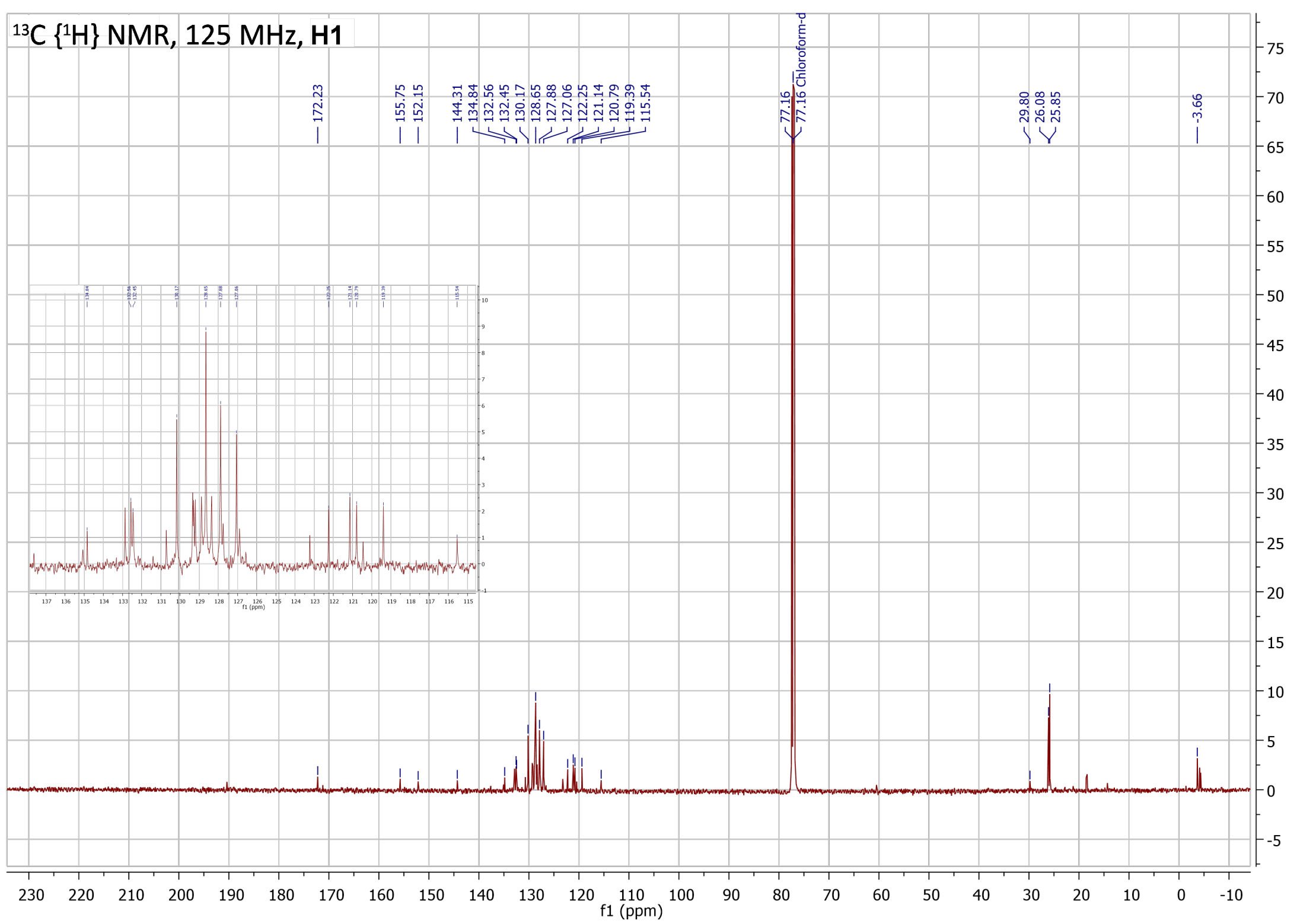

Figure S12: $^{1}H$ (top) and $^{13}C\{^{1}H\}$ (bottom) NMR spectra for **H1** in $CDCl_3$.

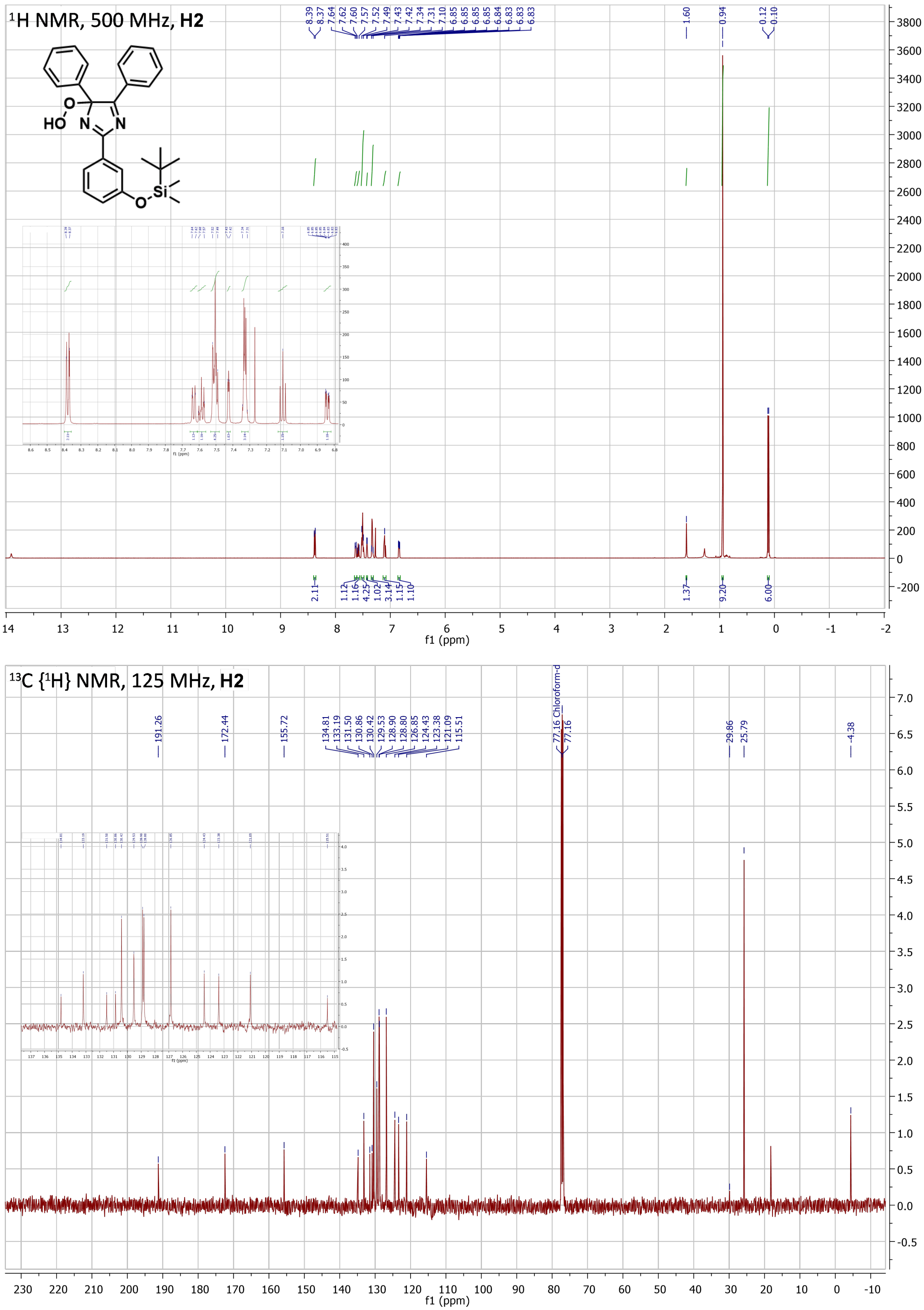


Figure S13: $^{1}H$ (top) and $^{13}C\{^{1}H\}$ (bottom) NMR spectra for **H2** in $CDCl_3$.

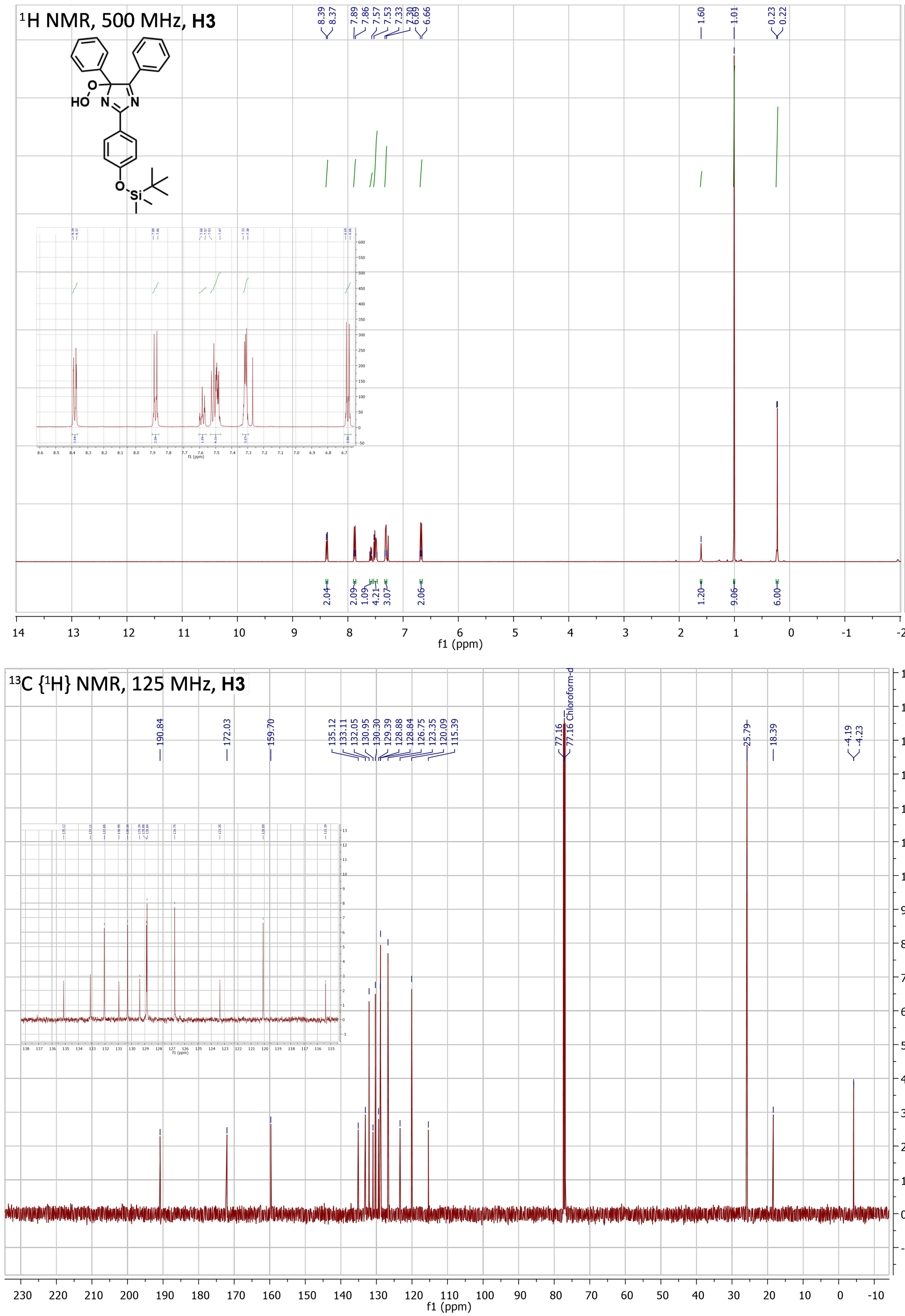


Figure S14: $^{1}H$ (top) and $^{13}C\{^{1}H\}$ (bottom) NMR spectra for **H3** in $CDCl_3$.

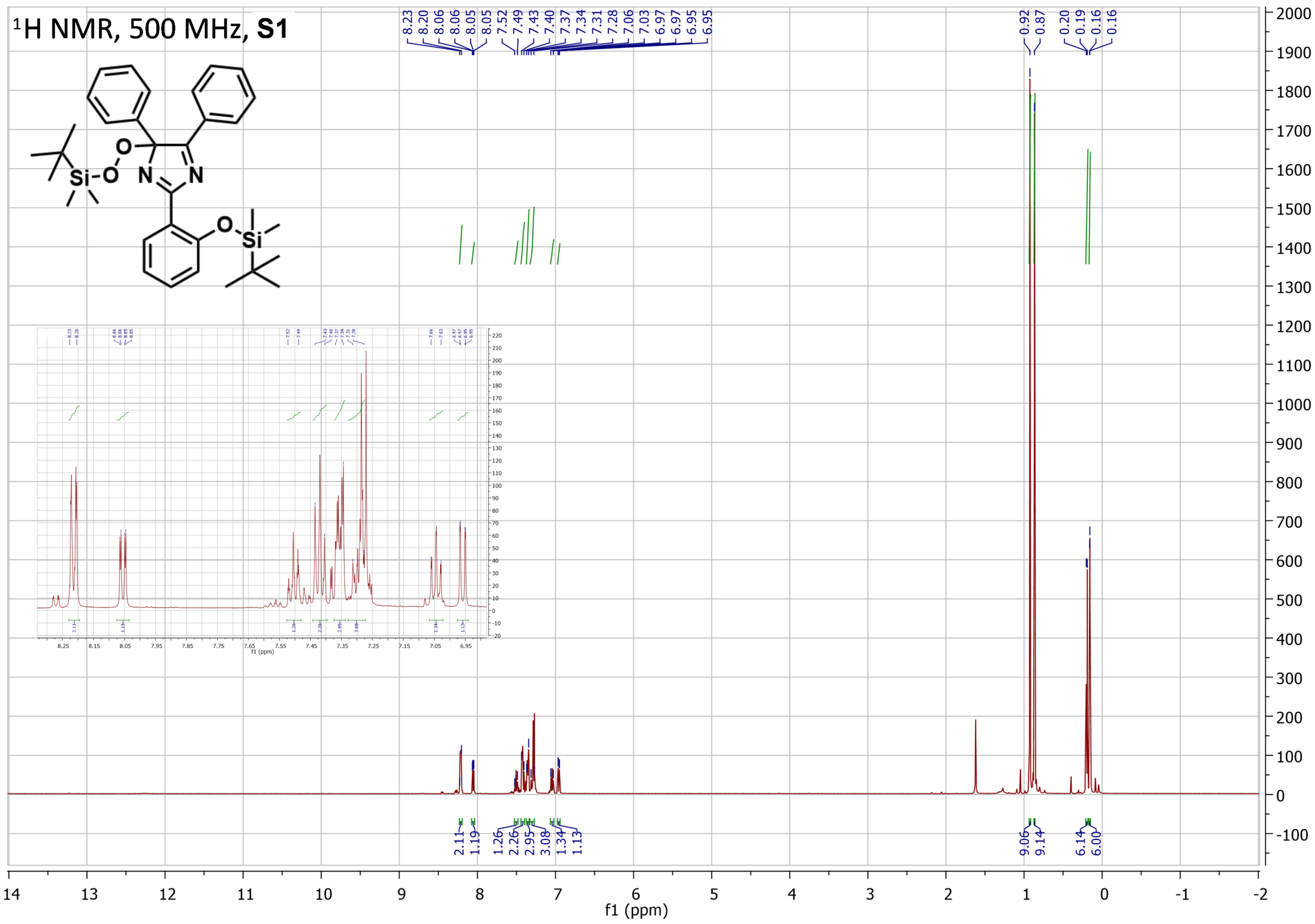


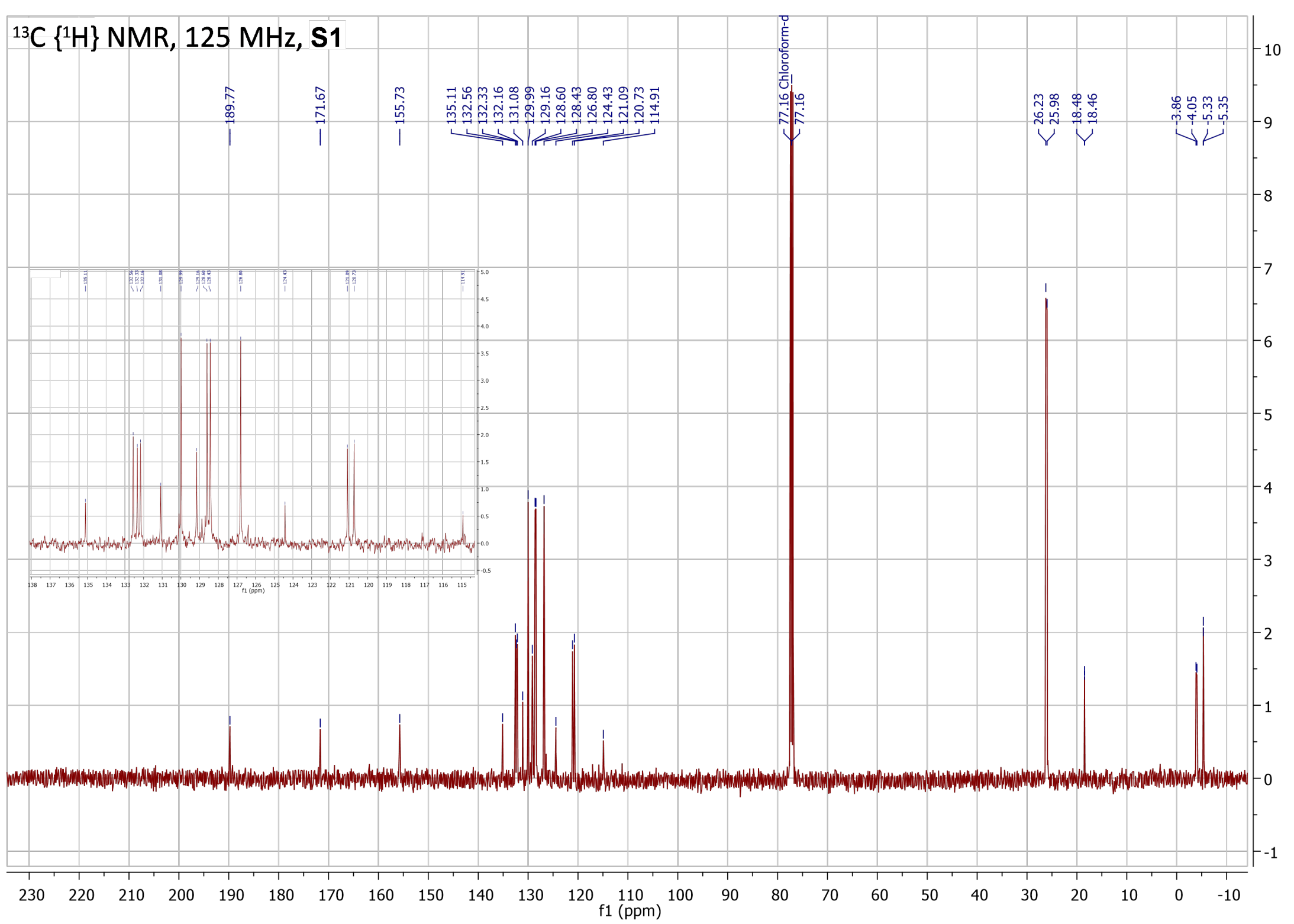


Figure S15: $^{1}$H (top) and $^{13}$C{$^{1}$H} (bottom) NMR spectra for **S1** in $CDCl_3$.

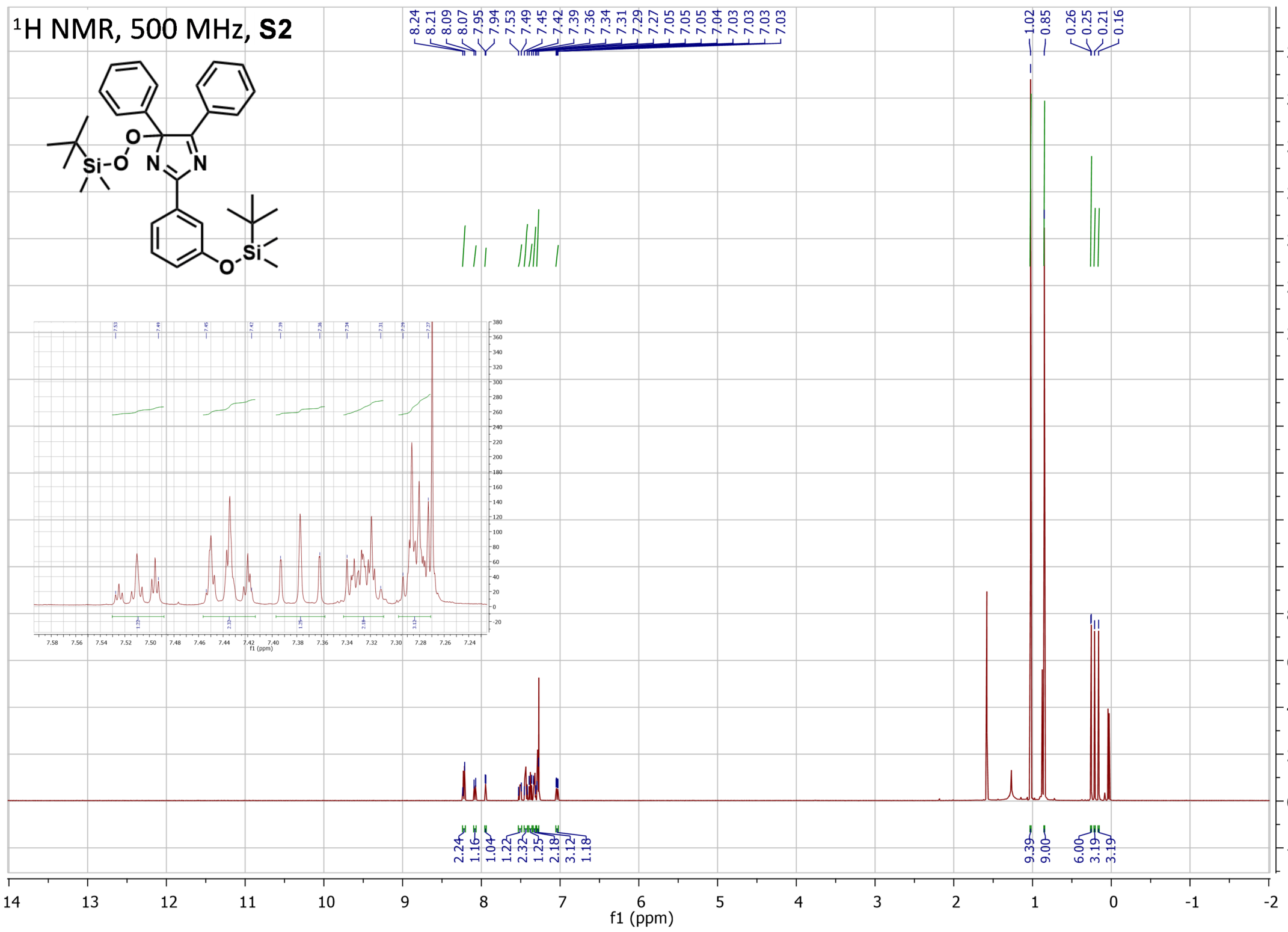


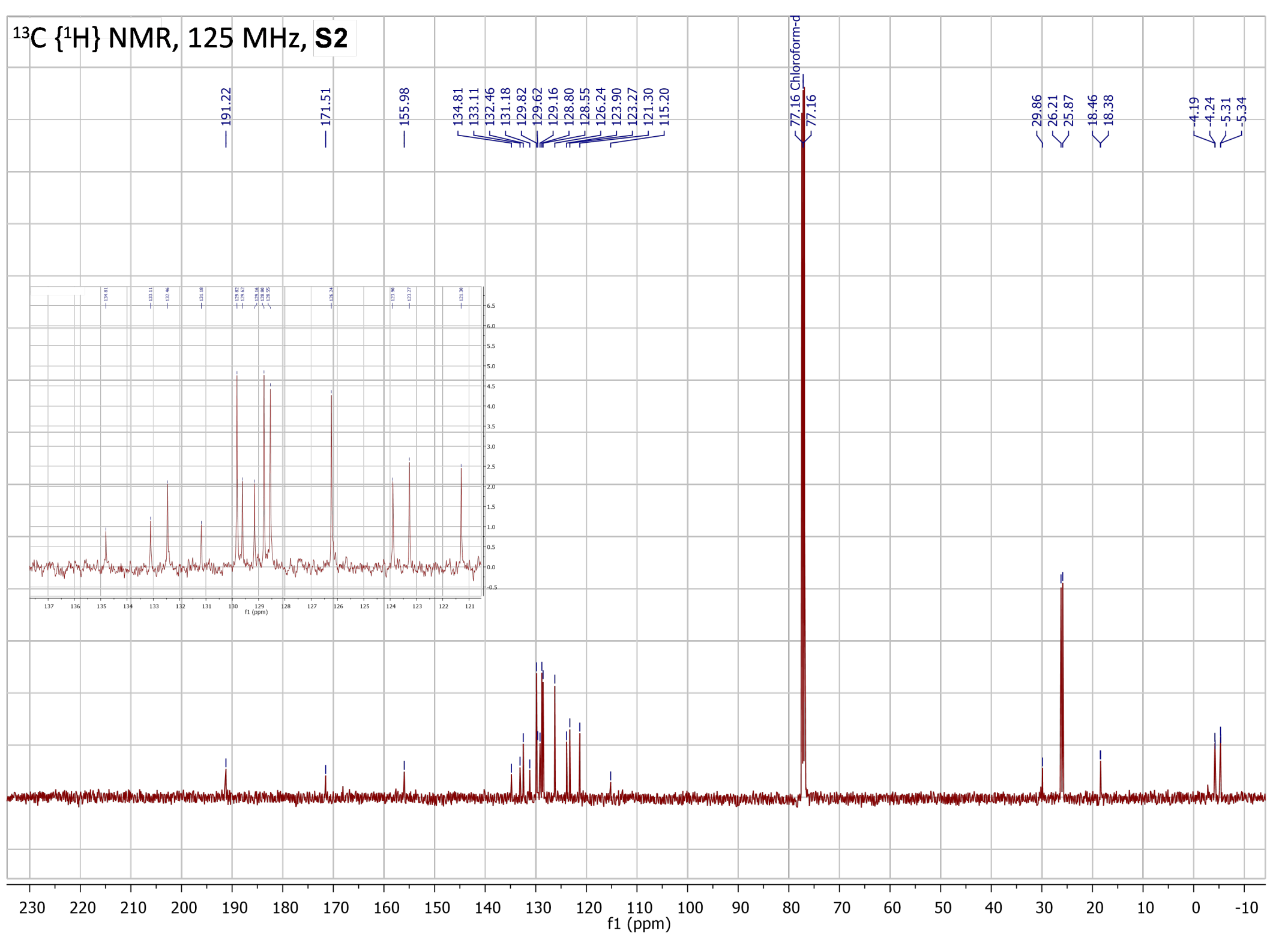


Figure S16: $^1$H (top) and $^{13}$C{$^1$H} (bottom) NMR spectra for **S2** in $CDCl_3$.

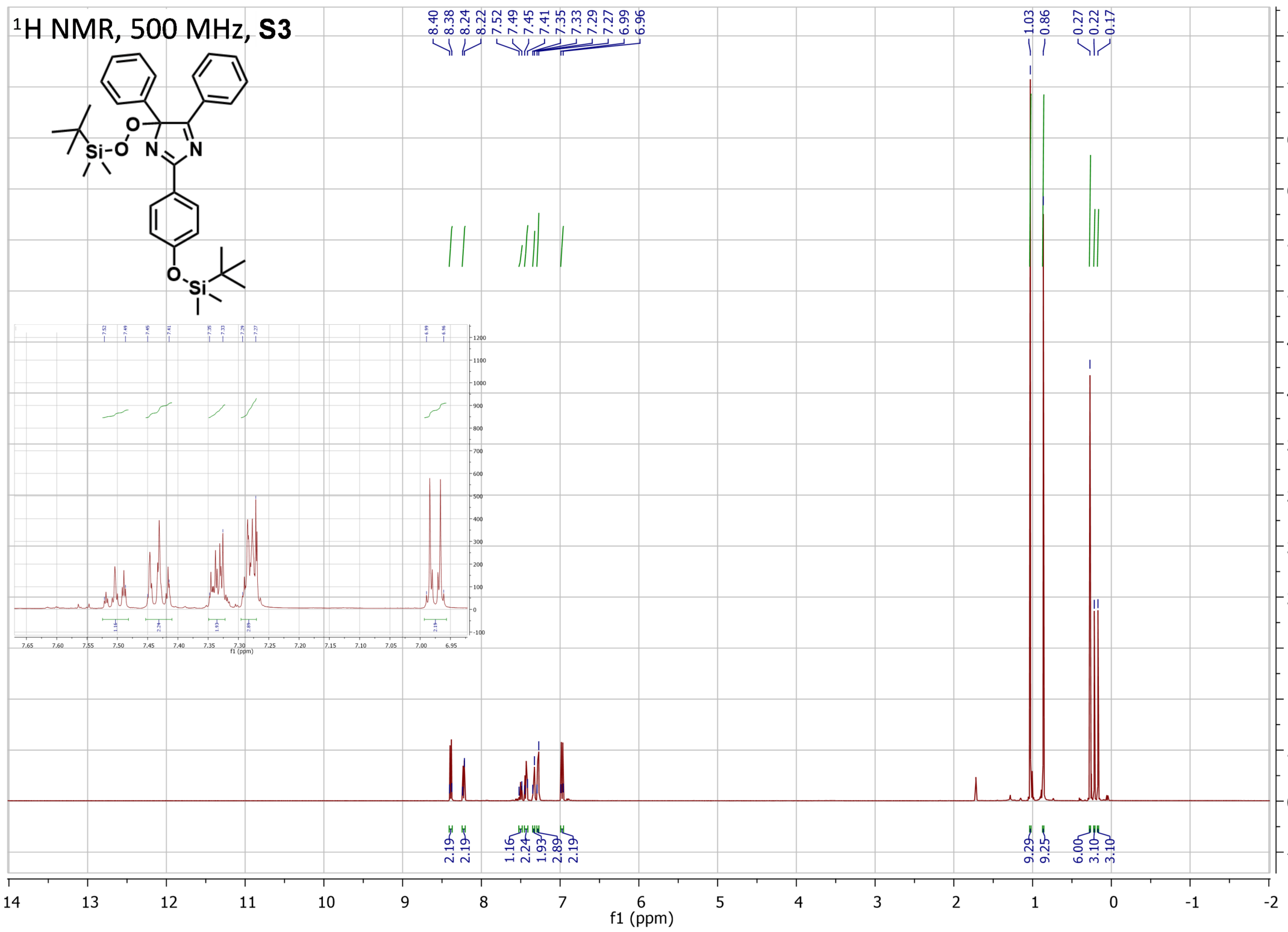


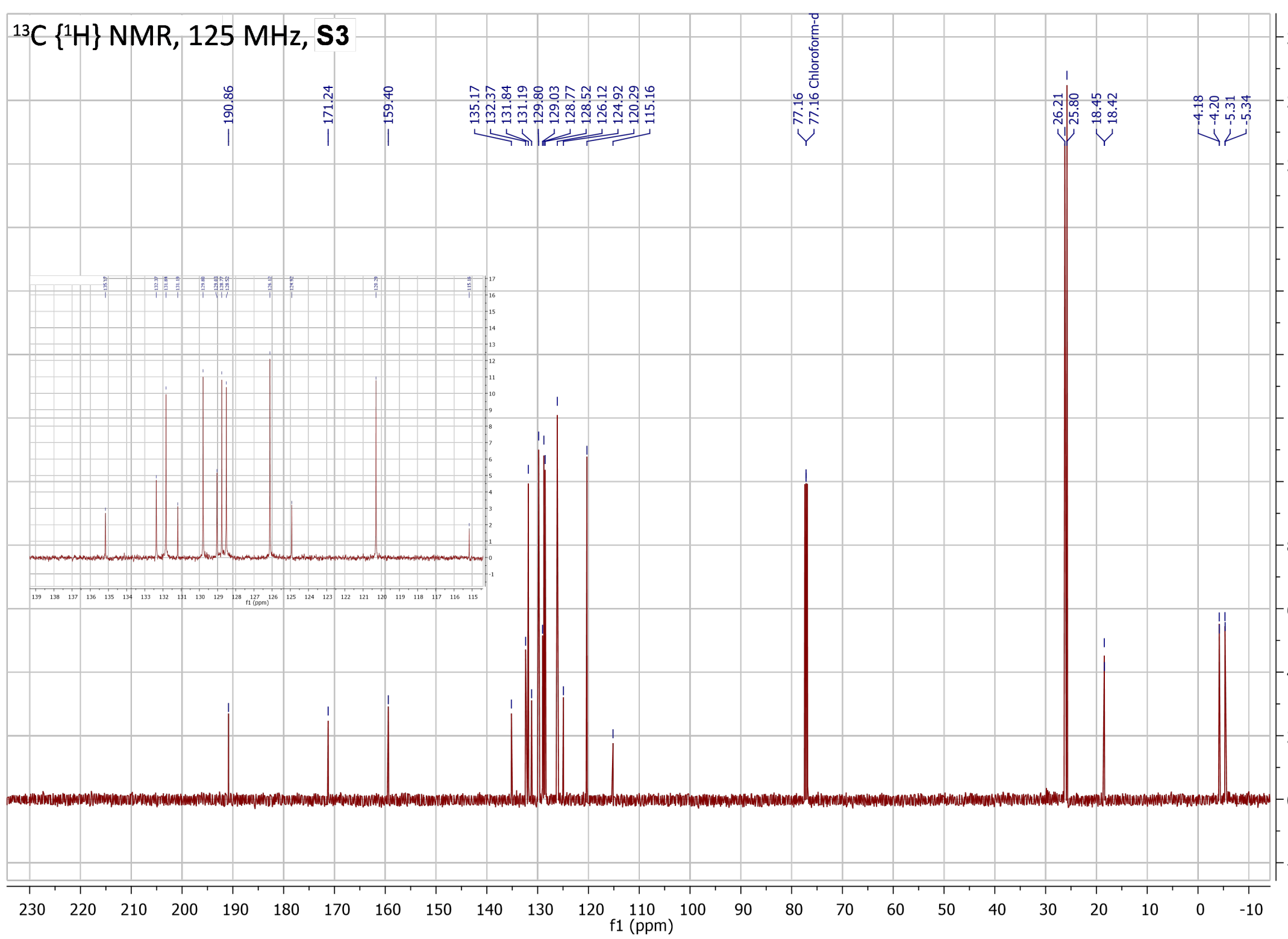


Figure S17: $^1$H (top) and $^{13}C\{^1H\}$ (bottom) NMR spectra for **S3** in $CDCl_3$.
